# MEG resting state alpha and beta band functional connectivity in male children with autism and sensory processing dysfunction

**DOI:** 10.1088/1741-2552/ae93fb

**Published:** 2026-08-28

**Authors:** Carly Demopoulos, Xuan Jesson, Molly Rae Gerdes, Barbora G Jurigova, Leighton B Hinkley, Kamalini G Ranasinghe, Shivani Desai, Anne Findlay, Srikantan S Nagarajan, Elysa J Marco

**Affiliations:** 1 Department of Psychiatry and Behavioral Sciences, University of California San Francisco, San Francisco, CA, United States of America; 2 Department of Radiology & Biomedical Imaging, University of California-San Francisco, San Francisco, CA, United States of America; 3 Department of Psychology, Palo Alto University, Palo Alto, CA, United States of America; 4 Cortica Healthcare, Department of Neurodevelopmental Medicine, San Rafael, CA, United States of America; 5 University of California-San Francisco, Department of Neurology, San Francisco, CA, United States of America; 6 Lifetime Neurodevelopmental Care, Inc, Department of Neurodevelopmental Medicine, Encino, CA, United States of America

**Keywords:** sensory processing, autism, imaginary coherence, magnetoencephalography

## Abstract

*Objective.* Sensory processing dysfunction (SPD) not only affects most individuals with autism spectrum disorder (ASD), but at least 5% of children without ASD also experience SPD. Our understanding of the relationship between sensory dysfunction and resting state brain activity is still emerging. The objective of this study was to examine group differences and behavioral associations with resting state alpha and beta oscillatory activity in ASD, SPD, and typically developing control (TDC) groups. *Approach.* This study compared long-range resting state functional connectivity of neural oscillatory behavior in 60 male children aged 8–12 years with (ASD; *N* = 18), those with (SPD; *N* = 18) who did not meet ASD criteria, and typically developing control participants (TDC; *N* = 24) using magnetoencephalography. Functional connectivity analyses were performed in the alpha and beta frequency bands, which are known to be implicated in sensory information processing. Group differences in functional connectivity and associations between sensory abilities and functional connectivity were examined. *Main results.* Distinct patterns of functional connectivity differences between ASD and SPD groups were found only in the beta band, but not in the alpha band. In both alpha and beta bands, ASD and SPD cohorts differed from the TDC cohort. Distinct patterns of associations between imaginary coherence and performance-based measures of sensory processing and verbal abilities were identified across groups. *Significance.* These findings demonstrate distinct long-range alpha and beta band phase-lagged neural synchrony alterations in SPD and ASD that are associated with sensory processing abilities in male children. These measures could serve as potential candidate neurophysiological markers for ASD and SPD at the group level, and may provide mechanistic insights relevant to biomarker development.

## 1. Introduction

Sensory dysfunction is estimated to impact at least 70% of individuals with autism spectrum disorders (ASDs) (Greenspan and Wieder 1997, Mayes and Calhoun 1999, Adamson *et al* 2006, Tomcheck and Dunn 2007, Al-Heizan *et al* 2015), and with its recognition as a core symptom in DSM-5 (American Psychiatric Association 2013), there is a rapidly growing body of research focused on understanding the causes and impact of sensory dysfunction in ASD. This line of research can be advanced not only by studying sensory dysfunction in individuals with ASD and other clinical populations, but also through examination of the estimated >5% of non-autistic individuals who experience clinically significant sensory processing dysfunction (SPD) (Ahn *et al* 2004). Yet, despite the impairment in adaptive functioning associated with SPD, the absence of a recognized categorical diagnosis limits access to resources for research and treatment in affected individuals. Nevertheless, biological differences, such as white matter abnormalities (Owen *et al* 2013, Chang *et al* 2014) and cortical response latencies (Demopoulos *et al* 2017a), have been identified in children with SPD and these measurable structural and physiologic differences have been associated with sensory processing behaviors (Chang *et al* 2016). While some features of sensory dysfunction may be shared among children with SPD and those with ASD, such as tactile processing deficits (Demopoulos *et al* 2015a), some domains of sensory dysfunction may identify important distinctions between these populations. For example, auditory processing abnormalities have been identified as distinguishing ASD from SPD groups in both behavioral tasks and neural response latencies (Demopoulos *et al* 2015a, 2017b). Understanding these similarities and differences in SPD among children with and without ASD can not only help delineate the sensory dysfunction that is specific to ASD, but it can also heighten our understanding of sensory information processing more broadly and guide treatment strategies.

Because differences in resting state oscillatory activity can be indicative of functional pathology (Papanicolaou 2009), there has been extensive research examining differences in resting state brain activity in individuals with and without ASD diagnoses. While previous sensory processing research has focused on differences in performance-based measures of, and neural responses to, sensory processing (Chang *et al* 2014, Demopoulos *et al* 2015a, 2017a, 2017b), our understanding of the relationship between sensory dysfunction and resting state brain activity is still emerging. This study will be the first to use silently acquired recording via magnetoencephalography (MEG) to examine whole brain functional connectivity during rest in participants with ASD, SPD, and typically developing control (TDC) participants. The goal of this study is to identify relevant differences in whole brain functional connectivity in the alpha and beta bands that may be associated with sensory dysfunction that is, versus is not, associated with autism. Concurrent examination of these three groups offers two key benefits. First, it will add to the emerging literature identifying the shared and distinct patterns of neural activity in children with ASD and SPD. Second, it will allow us to examine differences in functional connectivity and behavioral measures of sensory discrimination in affected children. Prior research has suggested that auditory and tactile processing are particularly impacted in children with ASD (Fernandez-Andres *et al* 2015), and that auditory processing has been associated with the language and nonverbal vocal communication impairments that are prevalent in ASD (Oram-Cardy *et al* 2005, Eigsti and Schuh 2008, Oram Cardy *et al* 2008, Roberts *et al* 2008, 2010, 2011, 2012, 2019, Edgar *et al* 2013, 2014, Lerner *et al* 2013, Demopoulos *et al* 2015a, 2015b, 2017a, 2017b, 2023a, 2023b, Demopoulos and Lewine 2016, Schwartz *et al* 2018, 2023, Arutiunian *et al* 2023, 2024, 2025, Li *et al* 2025, Gallo *et al* 2026). As such, we also examine associations between functional connectivity and performance-based measures of auditory and tactile processing and verbal abilities.

Our functional connectivity analyses were performed in the alpha and beta frequency bands, which are known to be implicated in sensory information processing. Specifically, these frequency bands have been associated with sensory gating (Buchholz *et al* 2014) and direction of sensory attention in the auditory and visual cortex for alpha (Foxe and Snyder 2011) and in the somatosensory cortex for beta (van Ede *et al* 2010, Bauer *et al* 2012). Further, the role of alpha activity in states of psychological distress has been widely studied (Boutcher and Landers 1998, Knyazev *et al* 2006, Fingelkurts *et al* 2007, Demerdzieva and Pop-Jordanova 2015, Smith *et al* 2016, Adolph and Margraf 2017, Mennella *et al* 2017), and may be relevant to differences in psychological response to sensory input in our clinical groups. While other frequency bands have also been associated with sensory processing (e.g. gamma), gamma band imaginary coherence estimates are more susceptible to muscle and movement artifacts. As such, we focused our investigation on the alpha and beta frequency bands.

Prior research has demonstrated that both children with SPD and ASD were impaired on behavioral and neural measures of tactile processing, but only the ASD group demonstrated auditory dysfunction (Demopoulos *et al* 2015a, 2017b). This work is consistent with structural findings that children with ASD and SPD demonstrate decreased connectivity in parieto-occipital tracts, but connectivity in temporal tracts was only reduced in the ASD group (Chang *et al* 2014). Thus, given these shared and divergent sensory findings between children with ASD and SPD, and given that alpha and beta connectivity has been associated with sensory gating and sensory attention in these frequency bands (Foxe and Snyder 2011, Buchholz *et al* 2014), we hypothesize that similar shared and divergent MEG-derived findings of resting state functional connectivity in the alpha and beta ranges will be identified between children with ASD, SPD, and TDC participants. In addition, based on work implicating alpha oscillations in the direction of auditory attention (Bauer *et al* 2012) and evidence of somatosensory cortex beta band modulation in advance of tactile stimuli (van Ede *et al* 2010), we also hypothesize that alpha connectivity will be associated with auditory processing and beta connectivity will be associated with tactile processing. To test these hypotheses, these frequency bands were subjected to source space reconstruction for analysis of differences in long-range phase-lagged neural synchrony in the alpha and beta bands, and associations with sensory processing abilities.

## 2. Methods

### 2.1. Participants

Participants were 60 boys aged 8–12 years (ASD *N* = 18; SPD *N* = 18; TDC *N* = 24) who were recruited from the UCSF Sensory Neurodevelopmental and Autism Program (SNAP) participant registry and website, UCSF pediatric neurology clinic, and local online parent groups. Experimental protocols were approved by the UCSF IRB (UCSF Committee on Human Research) and carried out in accordance with those approved procedures. Participants provided their written assent and written informed consent was obtained from parents or legal guardians prior to enrollment. Consent and assent procedures were witnessed by a member of the study team. Participants were recruited between 5/22/2003 and 10/26/2015. All participants who were taking medication were on a stable dose for at least six weeks prior to testing as reported in our previously published studies that recruited from this pool of participants (Demopoulos *et al* 2015a, 2017a). Specifically, in the TDC group one participant regularly used an antihistamine and a leukotriene inhibitor for seasonal allergies as well as melatonin for sleep. Another TDC participant regularly used steroid medications paired with a bronchodilator as needed for asthma and allergies and omeprazole for reflux. A third TDC participant regularly used methylphenidate for attention. In the SPD group, one participant was prescribed lisdexamfetamine, sertraline, and valproic acid for inattention and challenging behavior, and four others were taking stimulants (amphetamine/dextroamphetamine and methylphenidate) for inattention. One additional SPD participant was taking steroid medication for asthma. In the ASD group, one participant was taking a chelation agent (DMSA), another participant was taking escitalopram for anxiety, and a third was taking nonstimulant medication (atomoxetine) for attention and a leukotriene inhibitor for allergies.

Inclusion/exclusion criteria and diagnostic classification followed the criteria utilized in previous studies (Demopoulos *et al* 2015a, 2017a). Specifically, exclusion criteria included (1) bipolar disorder, psychotic disorder, or other neurological disorder or injury, and (2) a score of 70 or below on the Wechsler Intelligence Scale for Children-Fourth Edition (WISC-IV) (Wechsler 2003) Perceptual Reasoning Index (PRI). The PRI rather than the Full Scale Intelligence Quotient (FSIQ) was utilized for exclusion criteria because verbal abilities (represented in the Verbal Comprehension Index and incorporated into the FSIQ) were examined as an outcome measure in this study. Those with prior clinical diagnosis of ASD and those scoring ⩾15 on the Social Communication Questionnaire (SCQ, Rutter *et al* 2003), regardless of previous diagnostic status, were evaluated with the Autism Diagnostic Inventory-Revised (ADI-R) (Lord *et al* 1994) and the Autism Diagnostic Observation Schedule (ADOS) (Lord *et al* 1989). Diagnostic cutoffs on both of these measures were met for participants in the ASD group, who also met DSM-IV-TR criteria for Autistic Disorder, confirmed by a pediatric neurologist (EJM). Notably, sensory dysfunction was not included in the DSM-IV-TR diagnostic criteria for autistic disorder, whereas in the current DSM-5, sensory dysfunction is among the defining criteria of ASD. This may impact the inclusion of children into the ASD group (e.g. if they were lacking in other DSM-IV-TR B Criteria, clinically significant sensory dysfunction might have made the difference in meeting criteria according to DSM-5 standards). To ensure distinction from the SPD group, SPD participants were required to have been previously diagnosed with SPD by a community occupational therapist, were required to have SCQ score <15, and were required to score in the ‘Definite Difference’ range in one or more of the auditory, visual, oral/olfactory, tactile, vestibular, or multisensory processing domains of the Sensory Profile (Dunn 1999). All SCQ and Sensory Profile scores for the TDC group were not in clinical ranges. Because attention-deficit/hyperactivity disorder (ADHD) can co-occur in both autism and SPD, ADHD was not exclusionary. Our lower limit for participant age was based on prior work demonstrating that a reliable cortical auditory response can be elicited by age 8 (Shibasaki and Miyazaki 1992). The upper limit was determined according to availability of normative data for the DSTP, which goes up to 12 years and 11 months. Prior autism studies examining resting state neural oscillatory behavior have also restricted analyses to males given the higher prevalence of ASD in males (Zeidan *et al* 2022) as well as sex differences in peak alpha frequency (Edgar *et al* 2019, Green *et al* 2022, Manyukhina *et al* 2022). Because alpha connectivity was a focus for this project, only males participants were included to control for confounding effects of sex on oscillatory behavior in the alpha range. Demographic characteristics of the study sample are presented in table 1.

**Table 1.** Group characteristics (M ± SD [range]).

	ASD	SPD	TDC	Statistics
Age	9.88 ± 1.32 [8.13–12.00]	9.94 ± 1.29 [8.28–12.08]	10.18 ± 1.13 [8.18–11.94]	*F*(2,57) = .36
FSIQ	96.94 ± 13.54^a^^c^ [71–121]	109.39 ± 11.35 [89–131]	114.92 ± 9.31 [97–135]	*F*(2,57) = 13.20***
PRI	103.17 ± 8.56^d^ [94–123]	113.11 ± 11.63 [92–131]	111.00 ± 12.29 [89–129]	*F*(2,57) = 4.09*
VCI	98.56 ± 21.81^b^^d^ [59–140]	113.11 ± 14.63 [83–136]	118.46 ± 13.08 [93–144]	*F*(2,57) = 7.65**
Sensory Profile Total Score	135.78 ± 16.92^a^^c^ [102–160]	119.11 ± 17.38^a^ [74–145]	172.04 ± 10.38 [145–187]	*F*(2,57) = 71.12***
White (N)	10	12	17	
Asian (N)	4	1	1	
Multiracial (N)	4	3	4	
Hispanic (N)	0	1	0	
Unknown Race/Ethnicity (N)	0	1	2	
Right Handed (N)	15	17	20	
Left Handed (N)	1	1	2	
Ambidextrous (N)	2	0	1	
Unknown Handedness (N)	0	0	1	

*p < .05.

**p < .01.

***p < .001.

a Significantly different from TDC at *p* < .001 following Bonferroni correction for multiple comparisons.

b Significantly different from TDC at *p* < .01 following Bonferroni correction for multiple comparisons.

c Significantly different from SPD at *p* < .01 following Bonferroni correction for multiple comparisons.

d Significantly different from SPD at *p* < .05 following Bonferroni correction for multiple comparisons.

## 3. Measures

### 3.1. Tactile processing

Tactile processing measures were assessed according to previously published procedures (Demopoulos *et al* 2015a, 2017a). Tactile form discrimination was assessed using the Van Boven Domes (VBD) task (Van Boven and Johnson 1994, Van Boven Domes n.d.) and quantified by the lowest grating size of passed trials. Tactile proprioception was measured according to the total score of the right and left hand scores of the graphesthesia subtest of the Sensory Integration Praxis Tests (Ayres 1989).

### 3.2. Auditory processing

Auditory processing also was assessed according to previously published procedures (Demopoulos *et al* 2015a, 2017b) via the Acoustic (AI) and Acoustic-Linguistic Index (ALI) of the Differential Screening Test for Processing (DSTP) (Richard and Ferre 2006). The AI is derived from performance on measures of dichotic listening, temporal sequencing, and auditory filtering skills. The ALI assesses auditory processing skills associated with language via tasks focused on phonic and phonemic manipulation.

### 3.3. Verbal abilities

Because auditory processing dysfunction has been repeatedly associated with weaker verbal abilities in children with ASD (Oram-Cardy *et al* 2005, Russo *et al* 2009, Schmidt *et al* 2009, Roberts *et al* 2011, Edgar *et al* 2013, Demopoulos *et al* 2017b, 2023a, 2023b, Arutiunian *et al* 2023, 2025, Gallo *et al* 2026), we also assessed for associations between functional connectivity and verbal abilities in the ASD group using established protocols for our assessment of verbal abilities (Demopoulos *et al* 2015a, 2017b). The Linguistic Index (LI) of the DSTP was used to evaluate semantic and pragmatic aspects of language. The VCI of the WISC-IV (Wechsler 2003) was used to index verbal intellectual abilities (VIQ).

### 3.4. Magnetic resonance image (MRI) acquisition and processing

Structural MRIs were acquired for co-registration with MEG functional data on a 3 T Siemens MRI scanner at the UCSF Neuroscience Imaging Center. T1-weighted images were spatially normalized to the standard Montreal Neurological Institute template brain using 5 mm voxels in SPM8 (www.fil.ion.ucl.acuk/spm/software/spm8/). Normalization results were manually verified in all participants.

### 3.5. Magnetoencephalographic image acquisition and processing

Methods for acquisition and processing of MEG data follow protocols similar to those used in prior research employing these imaginary coherence metrics (Ranasinghe *et al* 2017, Demopoulos *et al* 2020). Specifically, MEG data were acquired at a 1200 Hz sampling rate using a 275-channel CTF System whole-head biomagnetometer (MEG International Services Ltd, Coqiotlam, BC, Canada). Fiducial coils were placed at the nasion and bilateral peri-auricular points to localize the head to the sensor array. These localizations were utilized for coregistration to the T1-weighted MRI and generation of a head shape. Four minutes of continuous recording was collected from each subject while awake with eyes closed in a supine position. While keeping eyes closed can increase alpha in resting state activity, it also serves to control visual stimulation and because this procedure was implemented for all participants, this would not confound group contrasts. As such, we elected to use an eyes closed approach, as has been used in many previous studies of resting state activity in children with ASD (J. Cornew *et al* 2012, Berman *et al* 2015, Edgar *et al* 2015b, 2019, Brodski-Guerniero *et al* 2018, Port *et al* 2019, Green *et al* 2020, 2022). Based on previous studies demonstrating reliable results from 60 s segments of MEG resting state data (Guggisberg *et al* 2008, Hinkley 2010, Hinkley *et al* 2011), we selected a 60 s artifact-free epoch. Artifact rejection criteria were signal amplitude >10 pT or visual evidence of movement or muscle contractions.

A whole brain lead field was computed according to a spatially normalized MRI with a 10 mm voxel size. The Neurodynamic Utility Tool for MEG (NUTMEG; http://nutmeg.berkeley.edu) (Dalal *et al* 2011) was used for source-space reconstruction and functional connectivity analyses. Source-space was reconstructed from filtered sensor data (fourth-order Butterworth filter of 1–20 Hz) using an adaptive beamformer. A linear combination of spatial weighting and sensor data matrices were used to estimate each voxel’s amplitudes (Hinkley *et al* 2011).

Following source space reconstruction, functional connectivity analysis was performed by computing imaginary coherence. The imaginary coherence approach excludes zero- or *π*-phase-lag-connectivity to eliminate neural synchrony attributable to volume spread (Nolte *et al* 2004). This approach has been documented as a reliable method for estimating long-range neural synchrony (Nolte *et al* 2004, Guggisberg *et al* 2008, Martino *et al* 2011, Engel *et al* 2013), and has been shown to reduce overestimation (Nolte *et al* 2004, Guggisberg *et al* 2008, Martino *et al* 2011). Imaginary coherence values were transformed to Fisher’s *Z* prior to calculating associations between each voxel and all other voxels. These associations were averaged within each voxel to derive values for voxel-wise mean imaginary coherence with all other voxels in the alpha and beta frequency bands. These values were examined for group contrasts and correlations with behavioral measures for the combined group study sample and separately for each group. All voxel-wise results with uncorrected *p* < 0.05 were further subjected to a 5% False Discovery Rate multiple comparisons correction (Benjamini and Hochberg 1995) and a 5-voxel cluster correction.

## 4. Missing data

Data from the sensory battery tasks are missing for some participants because these tasks were added to the protocol after these participants were enrolled. Thus, these data can be considered missing at random. DSTP data was available for 17 ASD participants, 17 TDC participants, and 11 SPD participants. Van Boven Domes were administered to 16 participants in the ASD group, 16 in the TDC group, and 11 in the SPD group. Graphesthesia was administered to 17 ASD participants, 15 TDC participants, and 11 SPD participants.

## 5. Results

Group contrasts and correlations with sensory processing and verbal abilities are summarized in the following sections with accompanying figures. Correlation analyses are presented for both the combined participant sample and for each group separately. Following these summarized results, tables 2–6 present a comprehensive list of all regions with statistically significant results following 5% FDR and 5-voxel cluster correction for group contrasts and correlation analyses.

**Table 2.** Summary of group contrast results.

Group	Band	Regions	Direction of difference
ASD vs TDC	*α*	Left fusiform and inferior occipital gyri and cerebellum	Decreased
	*α*	Right pre- and postcentral gyri	Increased
	*β*	Left middle and inferior temporal gyri	Decreased

SPD vs TDC	*α*	Left middle and superior frontal gyri and in the right inferior frontal gyrus, precuneus, and inferior and superior parietal lobules	Increased
	*β*	Bilaterally in the superior and middle frontal gyri, insula and putamen, as well as in the left inferior frontal gyrus, cingulate gyrus, caudate body, pre- and postcentral gyri, and inferior parietal lobule, and the right superior temporal gyrus, lentiform nucleus, globus pallidus, and caudate	Decreased

ASD vs SPD	*α*	No significant differences	N/A
	*β*	Right cingulate, middle frontal, and precentral gyri, and bilaterally in the superior and medial frontal gyri, the postcentral gyrus, the inferior parietal lobule, and in the supramarginal gyrus	Increased

**Table 3.** Summary of correlation results for the combine groups sample.

Band	Domain	Task	Regions	Direction of correlation
α	Tactile		No significant associations	N/A
α	Auditory	DSTP Acoustic Scale	Left cerebellar tonsil	+
α		DSTP Acoustic Scale	Left inferior and middle temporal gyri	—
α	Verbal	VIQ	Left uncus, cerebellar tonsil, and anterior superior, middle, and inferior temporal gyri	+

β	Tactile	Graphesthesia	Right precentral gyrus	—
β	Auditory		No significant associations	N/A
β	Verbal		No significant associations	N/A

**Table 4.** Summary of correlation results for the TDC group.

Band	Domain	Task	Regions	Direction of correlation
*α*	Tactile	Graphesthesia	Left superior, middle and inferior temporal gyri, parahippocampal gyrus, uncus, postcentral gyrus, inferior parietal lobule, supramarginal gyrus, cingulate, precuneus. Right superior, middle, and inferior frontal gyri, anterior cingulate, superior, middle, and inferior temporal gyri, postcentral gyrus, inferior parietal lobule	—
*α*	Auditory	DSTP Acoustic Scale	Left superior, middle and transverse temporal gyri, postcentral gyrus	—
*α*	Verbal	DSTP Linguistic Scale	Left inferior frontal gyrus and insula	+

*β*	Tactile	Graphesthesia	Right inferior and middle frontal and pre- and postcentral gyri, left middle frontal and precentral gyri	—
*β*	Auditory	DSTP Acoustic scale	Left middle, superior and transverse temporal gyrus, postcentral gyrus	—
*β*	Auditory	DSTP Acoustic/ Linguistic scale	Left cuneus, precuneus, middle occipital gyrus	—
*β*	Verbal		No significant associations	N/A

**Table 5.** Summary of correlation results for the ASD group.

Band	Domain	Task	Regions	Direction of Correlation
***α***	Tactile	Graphesthesia	Left insula, inferior, middle, and superior temporal gyri, the claustrum, the parahippocampal and fusiform gyri, the culmen, uncus, putamen, globus pallidus, caudate, hippocampus, midbrain; Right cingulate gyrus, middle and superior frontal gyri, insula, claustrum, putamen	+
*α*	Auditory	—	No significant associations	N/A
*α*	Verbal	—	No significant associations	N/A

*β*	Tactile	Van Boven Domes	Left middle frontal gyrus	—
*β*	Auditory	DSTP Acoustic scale	Left inferior and medial frontal gyri, anterior cingulate, middle and superior temporal gyri, and inferior parietal lobule; Right middle frontal gyrus, lingual gyrus, cerebellar declive, pyramis, and uvula	+
*β*	Verbal	VIQ	Left supramarginal, superior and middle temporal, fusiform, and angular gyri, inferior parietal lobule	+
*β*	Verbal	VIQ	Right cerebellar tonsil, inferior semilunar lobule, lentiform nucleus, globus pallidus, parahippocampal and fusiform gyri, middle and inferior temporal gyri	—

**Table 6.** Summary of correlation results for the SPD group.

Band	Domain	Task	Regions	Direction of Correlation
*α*	Tactile	Graphesthesia	Right postcentral gyrus, cuneus, precuneus, superior parietal lobule, cingulate, corpus callosum, pons, cerebellar tonsil, and culmen	+
*α*	Tactile	Van Boven Domes	Right cerebellar culmen and declive	—
*α*	Auditory	DSTP Acoustic/ Linguistic scale	Left postcentral and superior and middle temporal gyri	—
*α*	Verbal	VIQ	Bilateral inferior, middle and superior frontal gyri; Left inferior, middle and superior temporal gyri and fusiform, lingual, and parahippocampal gyri; Mesial middle occipital and posterior cingulate gyri, and cerebellar declive, uvula, pyramis, tuber, and inferior semilunar lobule	+
*α*	Verbal	DSTP Linguistic Scale	Right precentral and middle frontal gyri, amygdala, uncus	+

*β*	Tactile	Van Boven Domes	Right inferior frontal gyrus, thalamus, insula, and cingulate	+
*β*	Auditory	DSTP Acoustic/ Linguistic scale	Right inferior parietal lobule; Mesial precuneus/cingulate	+
*β*	Auditory	DSTP Acoustic/ Linguistic scale	Left superior and middle temporal gyri	—
*β*	Verbal	DSTP Linguistic scale	Left thalamus, midbrain, lingual gyrus, culmen, and declive	—

### 5.1. Group contrasts in imaginary coherence in the alpha band

**Figure 1.**
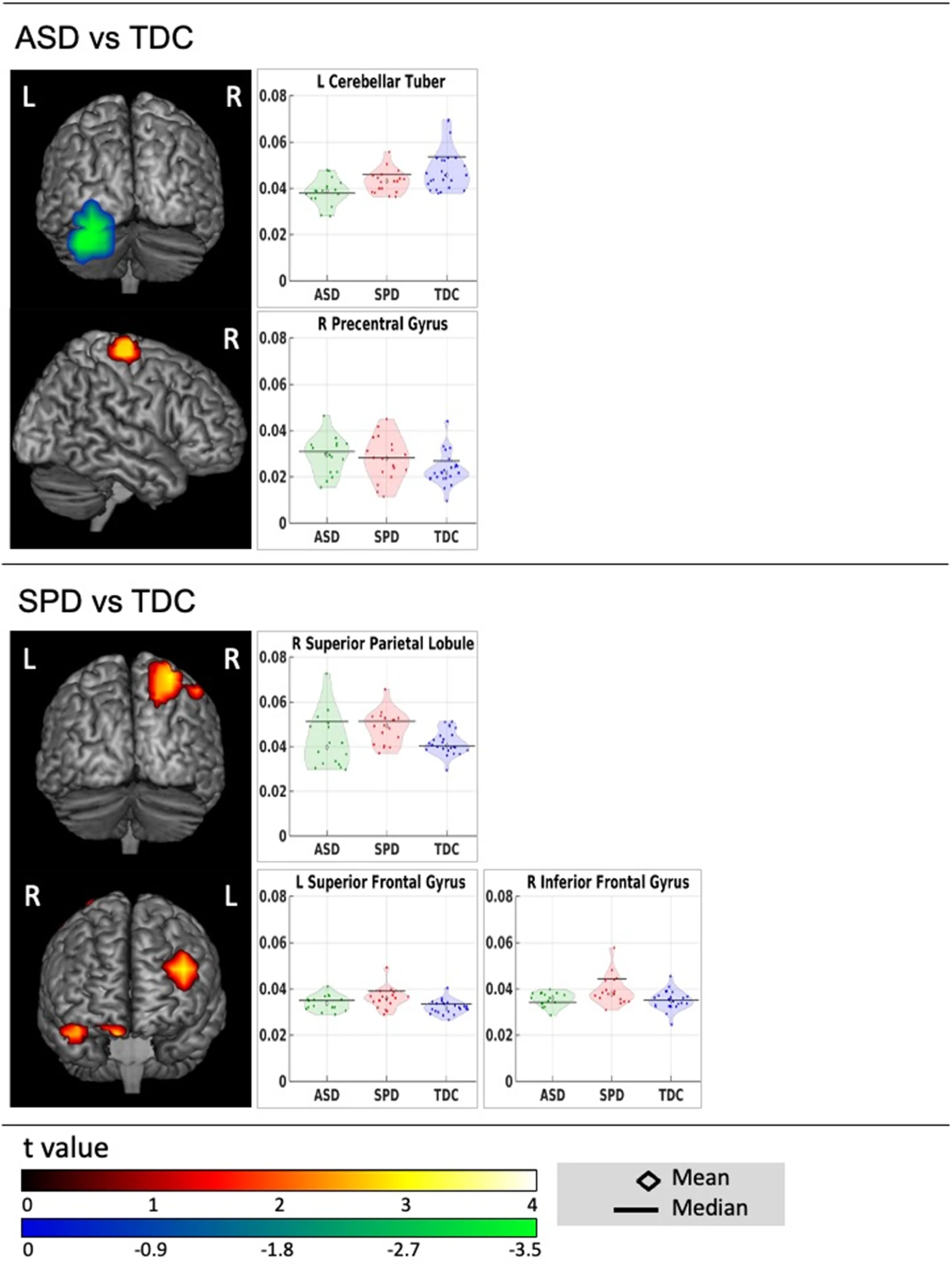
Group contrasts in alpha band imaginary coherence. Areas of significantly increased (warm) and reduced (cool) alpha imaginary coherence are presented for each pairwise contrast. Accompanying violin plots are presented showing imaginary coherence values for each group at the voxel within each cluster that demonstrated the greatest pairwise difference. Individual participant data points are presented on the violin plots as colored dots, with the mean represented as the black horizontal line, and the median represented as a black diamond.

Group contrasts in alpha coherence indicated that, relative to TDC participants, the ASD group showed reduced connectivity in the left fusiform and inferior occipital gyri and cerebellum and increased connectivity in the right pre- and postcentral gyri. No significant differences were identified between the ASD and SPD groups; however, the SPD group showed increased connectivity compared to TDC participants in the left middle and superior frontal gyri and in the right inferior frontal gyrus, precuneus, and inferior and superior parietal lobules. Alpha contrast results are presented in figure 1 and summarized in table 2. To ensure that these results were not driven by differences in medication usage across groups, we performed post hoc analyses of covariance controlling for the absence or presence of medication usage in each of the medication categories represented in our sample, including leukotriene inhibitors, antihistamines, melatonin, steroids, bronchodilators, proton-pump inhibitors, stimulants, SSRIs, anticonvulsants, SNRIs, and chelation agents. All statistically significant contrasts presented in figure 1 remained significant after controlling for medication usage.

### 5.2. Correlations between alpha imaginary coherence and sensory processing/verbal abilities

**Figure 2.**
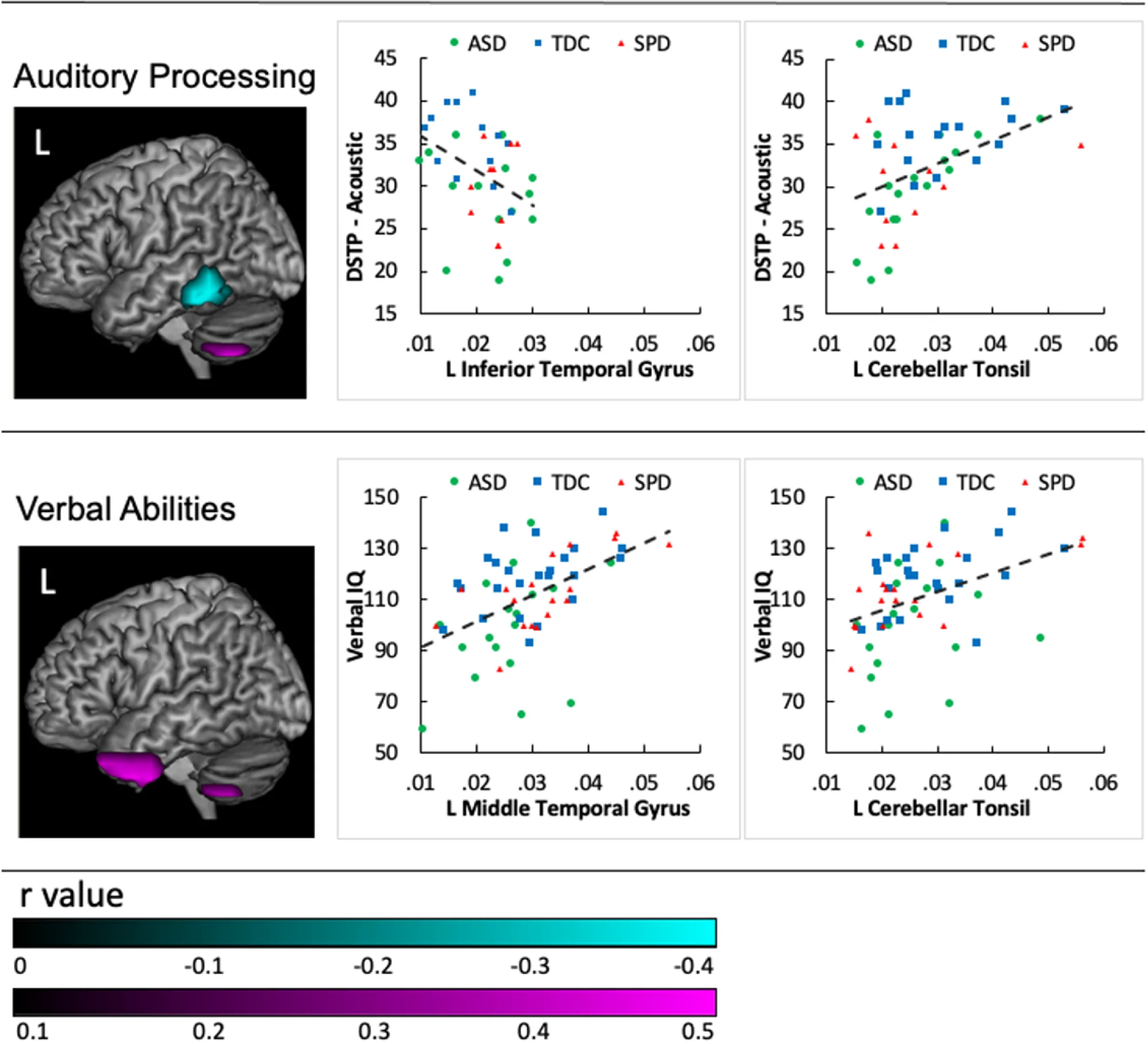
Correlations between clinical measures and alpha imaginary coherence in the combined participant sample. Positive associations between auditory processing/verbal abilities and alpha imaginary coherence values are identified in magenta clusters for the sample of all participants in the study. Negative associations between auditory processing and alpha imaginary coherence values are identified in cyan clusters. Corresponding scatterplots are presented for the voxel with the maximum correlation value within each cluster, with groups identified by color and shape (ASD group = green circle, SPD group = red triangle, and TDC group = blue square).

**Combined group alpha band correlations** Correlation analyses were performed on all study participants combined across groups to examine the relations between functional connectivity and the range of sensory processing and verbal abilities in our sample. No significant associations were identified between tactile processing performance and measures of alpha coherence; however, significant associations were identified between measures of alpha coherence and auditory processing performance. Specifically, scores on the DSTP Acoustic scale were positively associated with alpha coherence in the left cerebellar tonsil and negatively associated with alpha coherence in the left inferior and middle temporal gyri. A significant positive association also was identified between VIQ and alpha coherence in the left uncus, cerebellar tonsil, and anterior superior, middle, and inferior temporal gyri (figure 2). A summary of correlation results is presented in table 3. Again, to ensure that these associations were not an artifact of medication effects, we performed post hoc hierarchical regression analyses to determine if the statistically significant associations presented in figure 2 remained significant after controlling for medication usage. Presence or absence of medication usage in each medication category were included as independent variables in step 1 of the analysis. Functional connectivity values were included in step 2 and the performance-based measure of sensory or communication functioning was entered as the dependent variable. These results indicated that all associations between sensory or communication performance and functional connectivity were statistically significant after controlling for medication usage.

**Figure 3.**
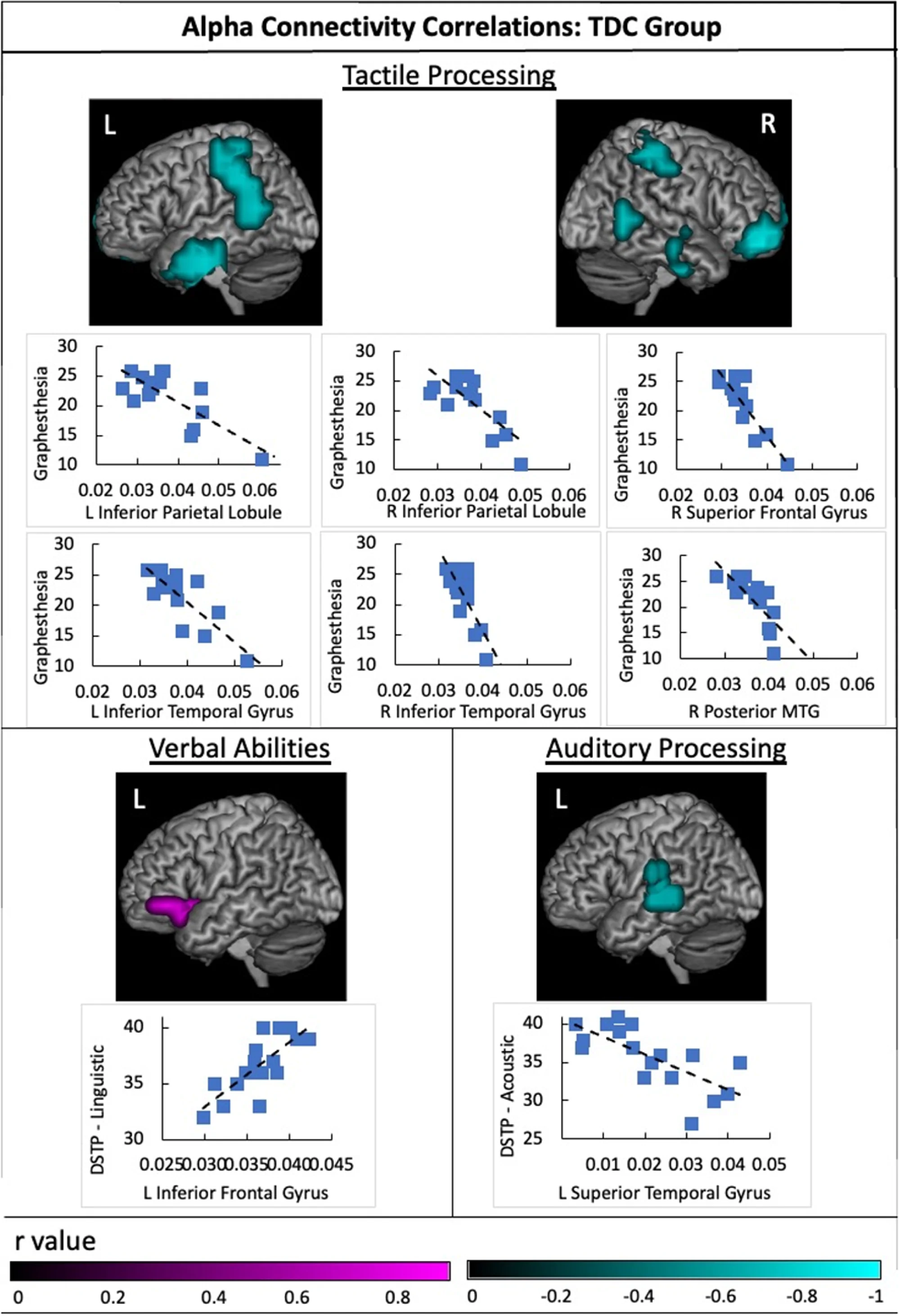
Correlations between clinical measures and alpha imaginary coherence in the TDC Group. Associations between sensory processing/verbal abilities and imaginary coherence values are identified in clusters (magenta for positive associations and cyan for negative associations). Corresponding scatterplots are presented for the voxel with the maximum correlation value within each cluster. Negative correlations were identified between sensory processing and imaginary coherence, with associations between auditory processing and a left temporal region and between tactile processing and a more distributed bilateral set of frontal, temporal and parietal regions. In contrast, linguistic performance was positively associated with left frontal imaginary coherence.

**TDC group alpha band correlations.** In the TDC group, performance on the Acoustic Scale of the DSTP was negatively associated with alpha connectivity in a left temporal region whereas DSTP Linguistic Scale performance was positively associated with alpha connectivity in the left inferior frontal gyrus/insula. Graphesthesia performance was negatively associated with alpha connectivity bilaterally. Specifically, negative associations were identified in bilateral temporal and parietal and right frontal regions (figure 3).

**Figure 4.**
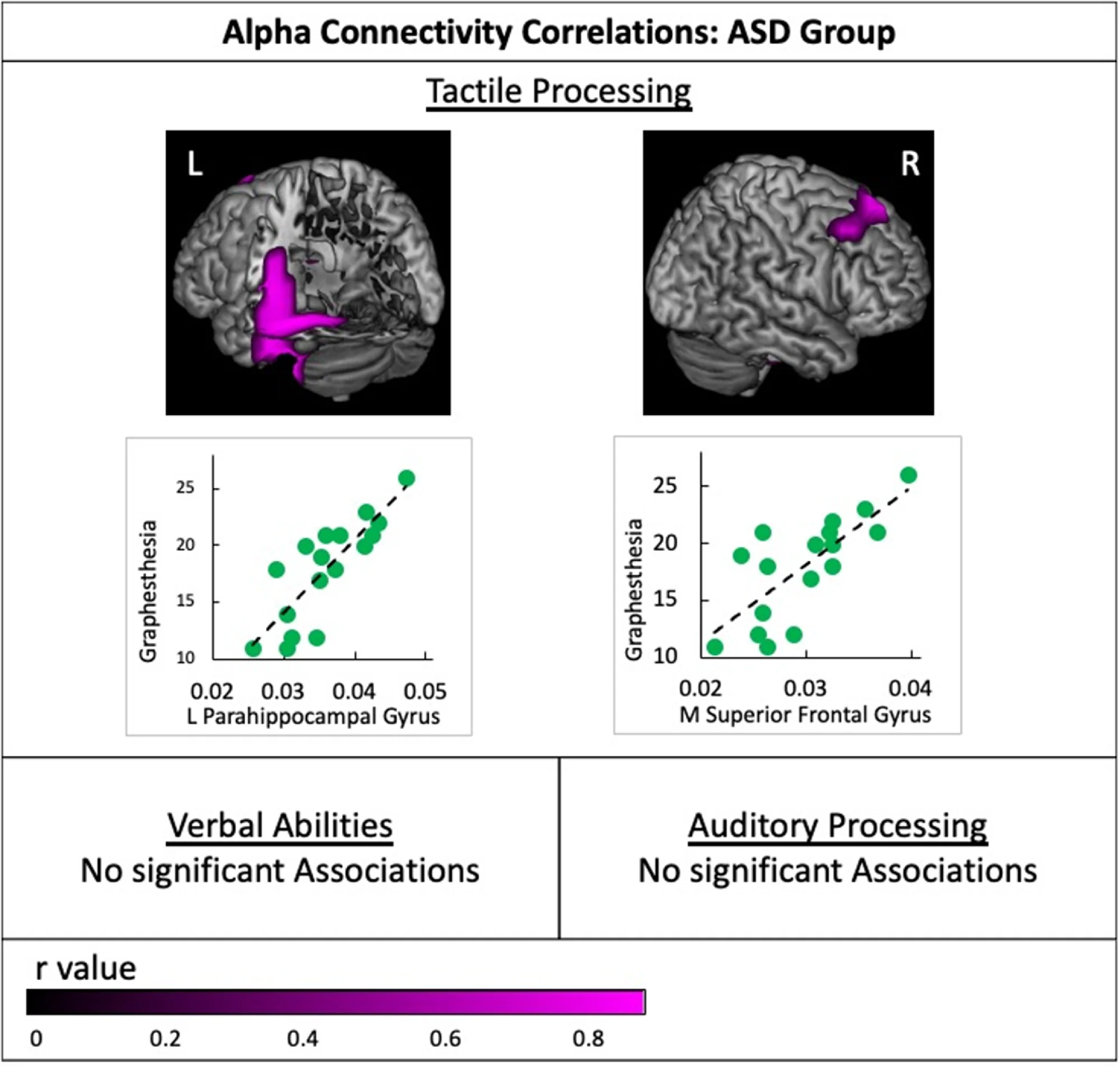
Correlations between clinical measures and alpha imaginary coherence in the ASD group. Positive associations between tactile processing on the graphesthesia task and imaginary coherence values are identified in magenta clusters. Corresponding scatterplots are presented for the voxel with the maximum correlation value within each cluster. Positive associations were identified between graphesthesia performance and both right frontal and left temporal and subcortical structures.

**ASD group alpha band correlations.** In contrast to the TDC group, no significant associations were identified between alpha connectivity and measures of auditory processing or verbal abilities in the ASD group. The ASD group presented with a pattern of positive associations between alpha connectivity and tactile processing. Specifically, graphesthesia scores were positively associated with connectivity in left temporal and subcortical structures and right frontal regions (figure 4).

**Figure 5.**
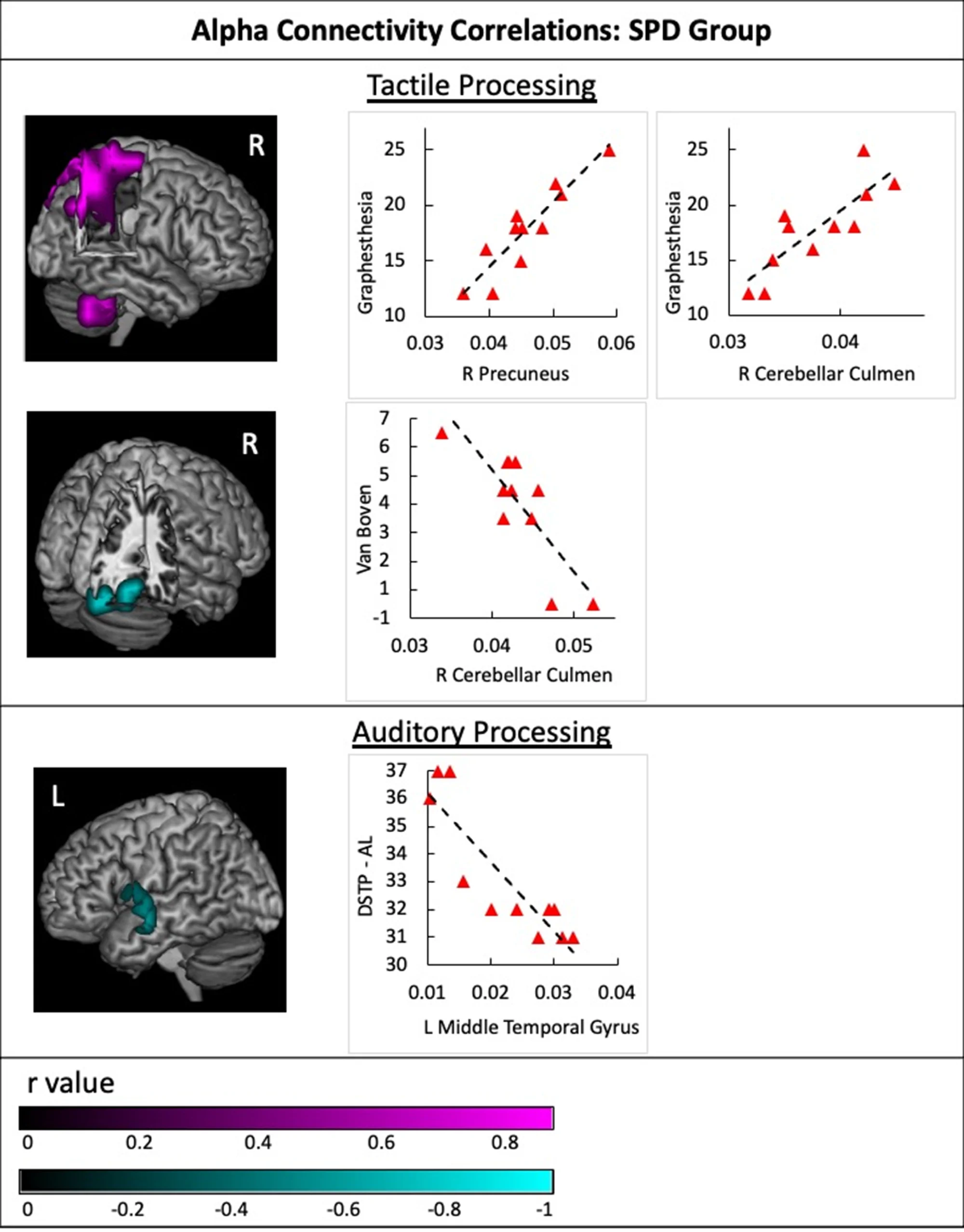
Correlations between sensory measures and alpha imaginary coherence in the SPD group. Associations between sensory processing and imaginary coherence values are identified in clusters (magenta for positive associations and cyan for negative associations). Corresponding scatterplots are presented for the voxel with the maximum correlation value within each cluster. Negative associations were identified between a left temporal region and auditory processing and a left cerebellar region and Von Boven Domes task performance. Positive associations were identified between graphesthesia performance and right cerebellar and parietal regions.

**Figure 6.**
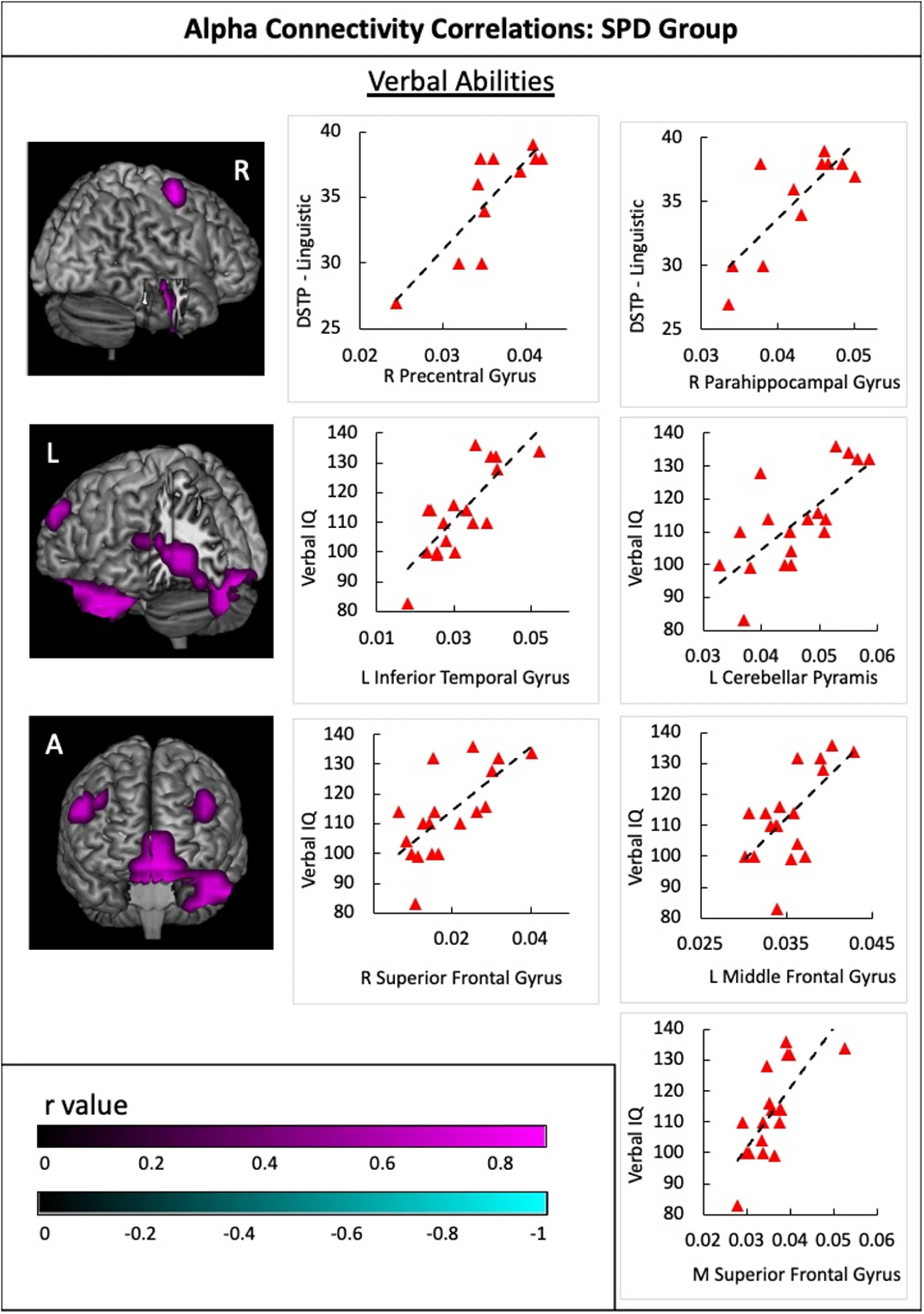
Correlations between verbal abilities and alpha imaginary coherence in the SPD group. Associations between verbal abilities and imaginary coherence values are identified in clusters (magenta for positive associations and cyan for negative associations). Corresponding scatterplots are presented for the voxel with the maximum correlation value within each cluster. Positive associations were identified between verbal abilities and a more distributed bilateral network of frontal, temporal, parietal, occipital, and cerebellar regions.

**SPD group alpha band correlations.** DSTP Acoustic Linguistic scores were negatively associated with left temporal alpha imaginary coherence. Both positive and negative associations were identified between tactile performance and right hemisphere alpha connectivity (figure 5). Specifically, graphesthesia scores were significantly associated with increased connectivity in the right parietal, subcortical, and cerebellar regions. In contrast, performance on the Von Boven Domes task was negatively associated with connectivity in right cerebellar regions. Moreover, alpha band imaginary coherence in multiple brain regions was significantly associated with verbal abilities. Specifically, in the right hemisphere, VIQ was positively associated with alpha connectivity in the precentral and middle frontal gyri and the pyramis. In the left hemisphere, VIQ was positively associated with alpha coherence in a distributed network of frontal, temporal, occipital and cerebellar structures. Performance on the DSTP Linguistic Scale was positively associated with alpha band imaginary coherence in the right precentral and middle frontal gyri, the amygdala, and the uncus (figure 6).

### 5.3. Group contrasts in imaginary coherence in the beta band

**Figure 7.**
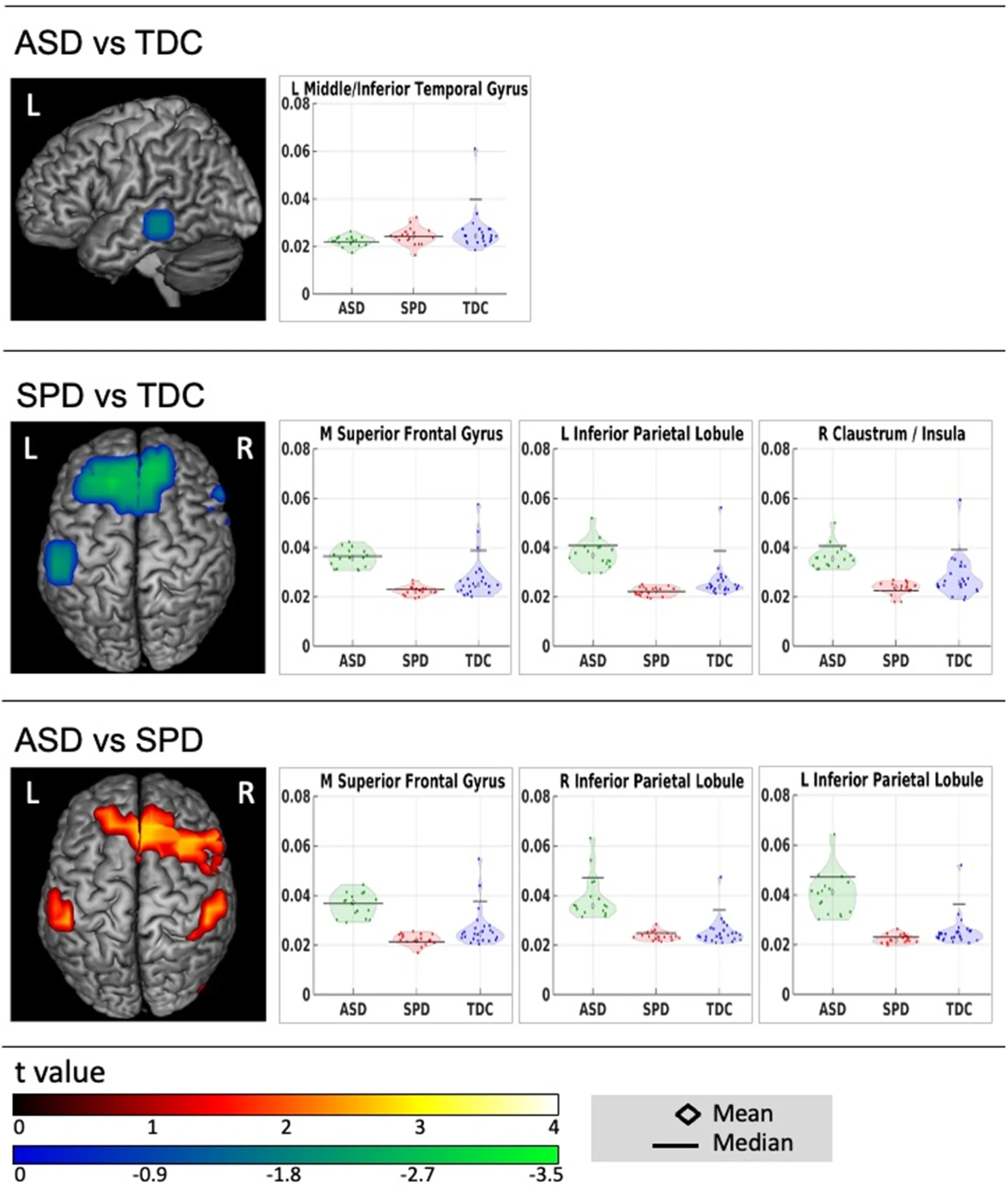
Group contrasts in beta band imaginary coherence. Areas of significantly increased (warm) and reduced (cool) beta imaginary coherence are presented for each pairwise contrast. Accompanying boxplots are presented showing imaginary coherence values for all groups at the voxel within each cluster that demonstrated the greatest pairwise difference. Notably, the contrast in left inferior parietal lobule beta connectivity between the SPD and TDC groups was no longer statistically significant after controlling for medication usage. Individual participant data points are presented on the violin plots as colored dots, with the mean represented as the black horizontal line, and the median represented as a black diamond.

Group contrasts in beta coherence indicated that, relative to TDC participants, the ASD group showed reduced imaginary coherence in the left middle and inferior temporal gyri. Relative to SPD participants, however, the ASD group showed a pattern of increased beta band imaginary coherence in the right and mesial frontal regions and bilateral inferior parietal lobules. Finally, when compared to TDC participants, the SPD group demonstrated a pattern of reduced beta connectivity bilaterally in left and mesial frontal regions, right insula, and left inferior parietal lobule. Beta contrasts are presented in figure 3 and summarized in table 2. All beta connectivity contrasts in figure 3 remained statistically significant after controlling for medication usage except for the contrast between the SPD and TDC groups in the left IPL (*p* = .052) (figure 7).

### 5.4. Correlations between beta imaginary coherence and sensory processing/verbal abilities

**Figure 8.**
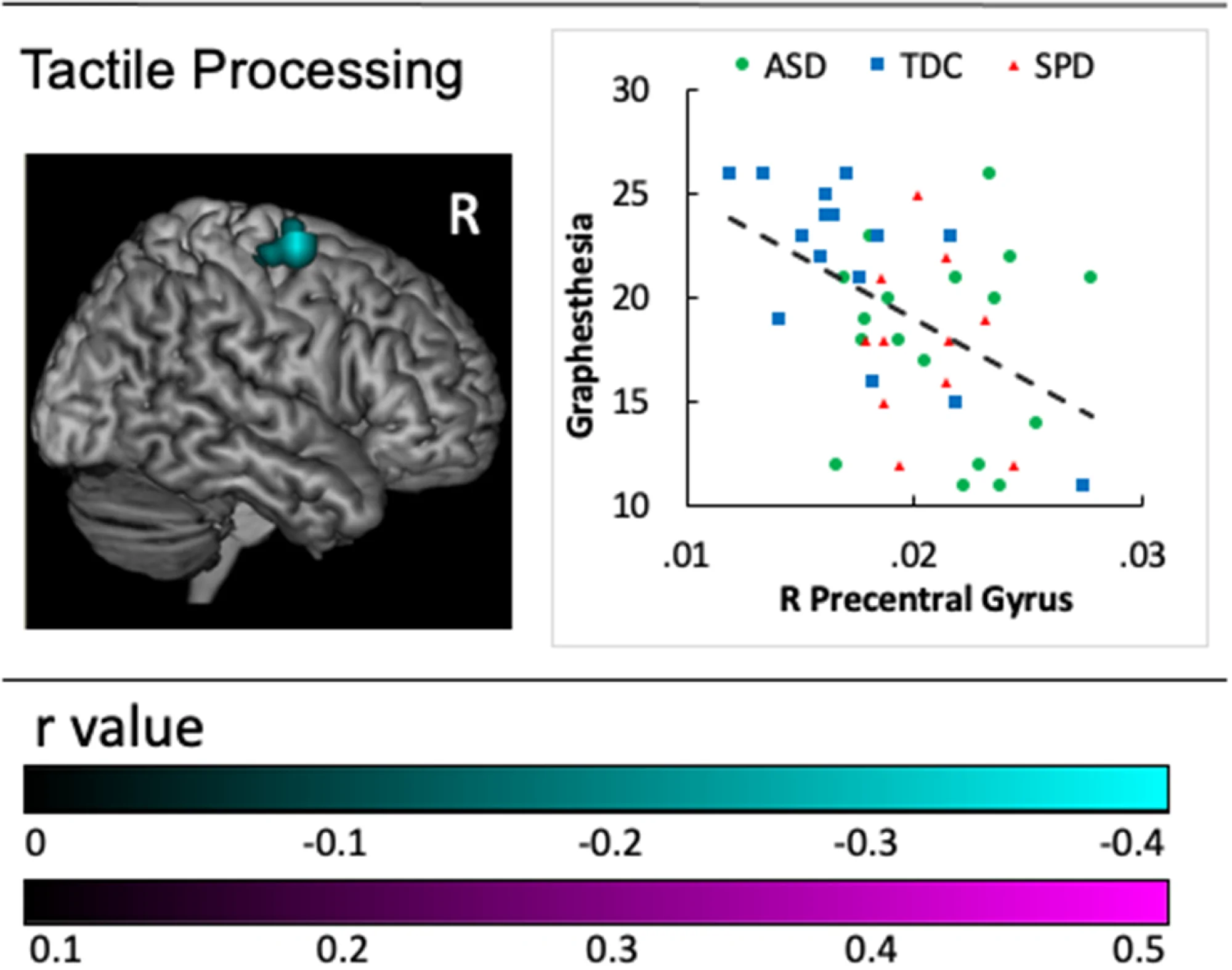
Correlations between clinical measures and beta imaginary coherence in the combined participant sample. Negative associations between tactile processing abilities and beta imaginary coherence values are identified in the cyan cluster for the sample of all participants in the study. The corresponding scatterplot is presented for the voxel with the maximum correlation value within each cluster, with groups identified by color and shape (ASD group = green circle, SPD group = red triangle, and TDC group = blue square).

**Combined group beta band correlations.** A significant negative association was identified between beta coherence in the right precentral gyrus and performance on the graphesthesia task (figure 4). This association (figure 4) remained statistically significant after controlling for the effects of medication usage. No significant associations were identified between beta coherence and measures of auditory processing or verbal abilities in the combined groups sample. A summary of combined group correlation results is presented in table 3 (figure 8).

**Figure 9.**
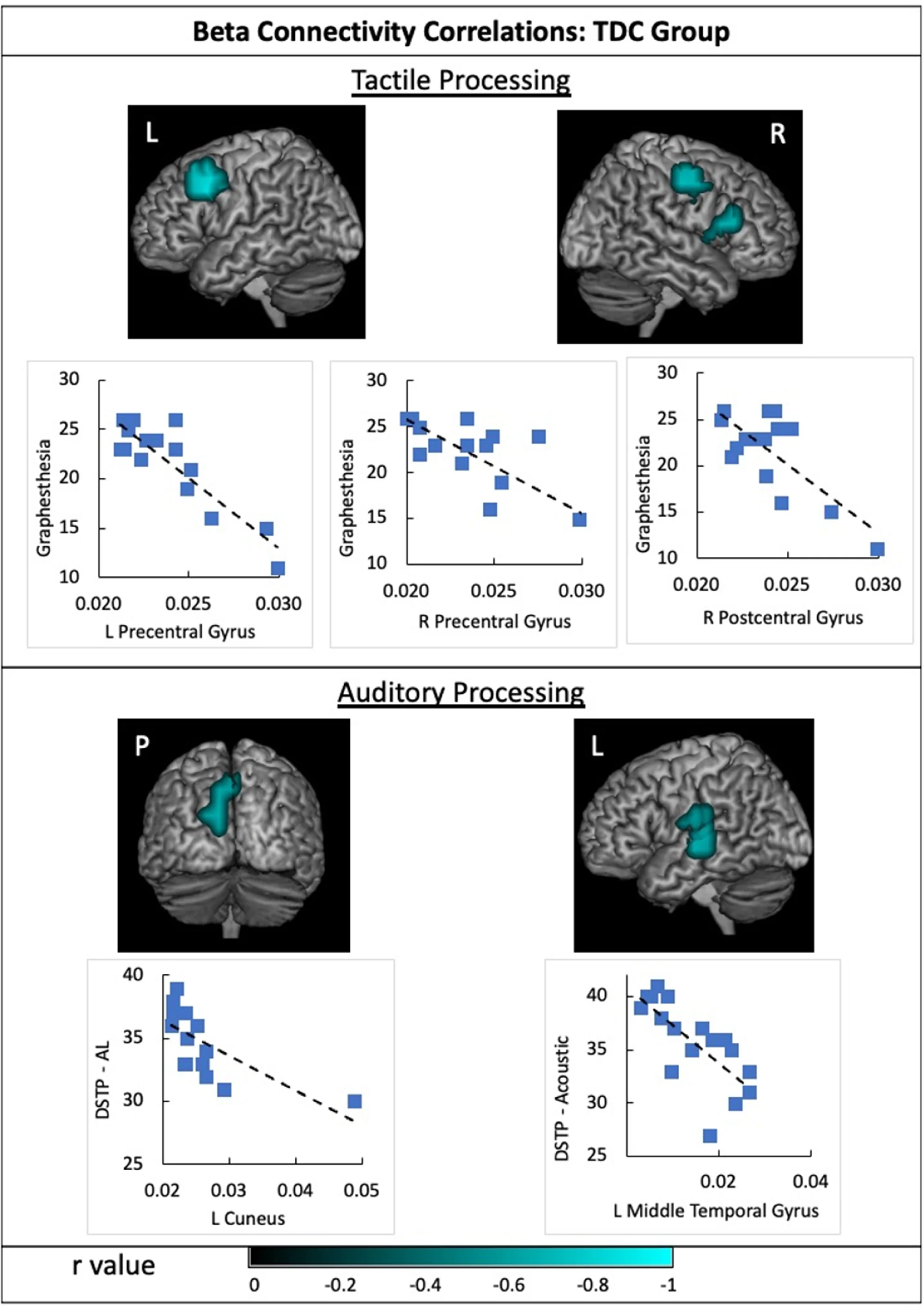
Correlations between clinical measures and beta imaginary coherence in the TDC group. Associations between sensory processing/verbal abilities and imaginary coherence values are identified in cyan clusters. Corresponding scatterplots are presented for the voxel with the maximum correlation value within each cluster. Negative correlations were identified between auditory processing and beta band imaginary coherence in left temporal and occipital regions and between tactile processing measures and left frontal and right frontal and parietal regions.

**TDC group beta band correlations.** Verbal abilities were not significantly associated with beta coherence in the TDC group; however, tactile processing on the graphesthesia task was negatively associated with beta connectivity bilaterally in frontal and sensorimotor cortices. Auditory processing was also negatively associated with beta connectivity. Specifically, negative association with the DSTP Acoustic scale were identified in left temporal and postcentral regions, and negative associations with the DSTP Acoustic/Linguistic scale were identified in the left parietal and occipital regions (figure 9).

**Figure 10.**
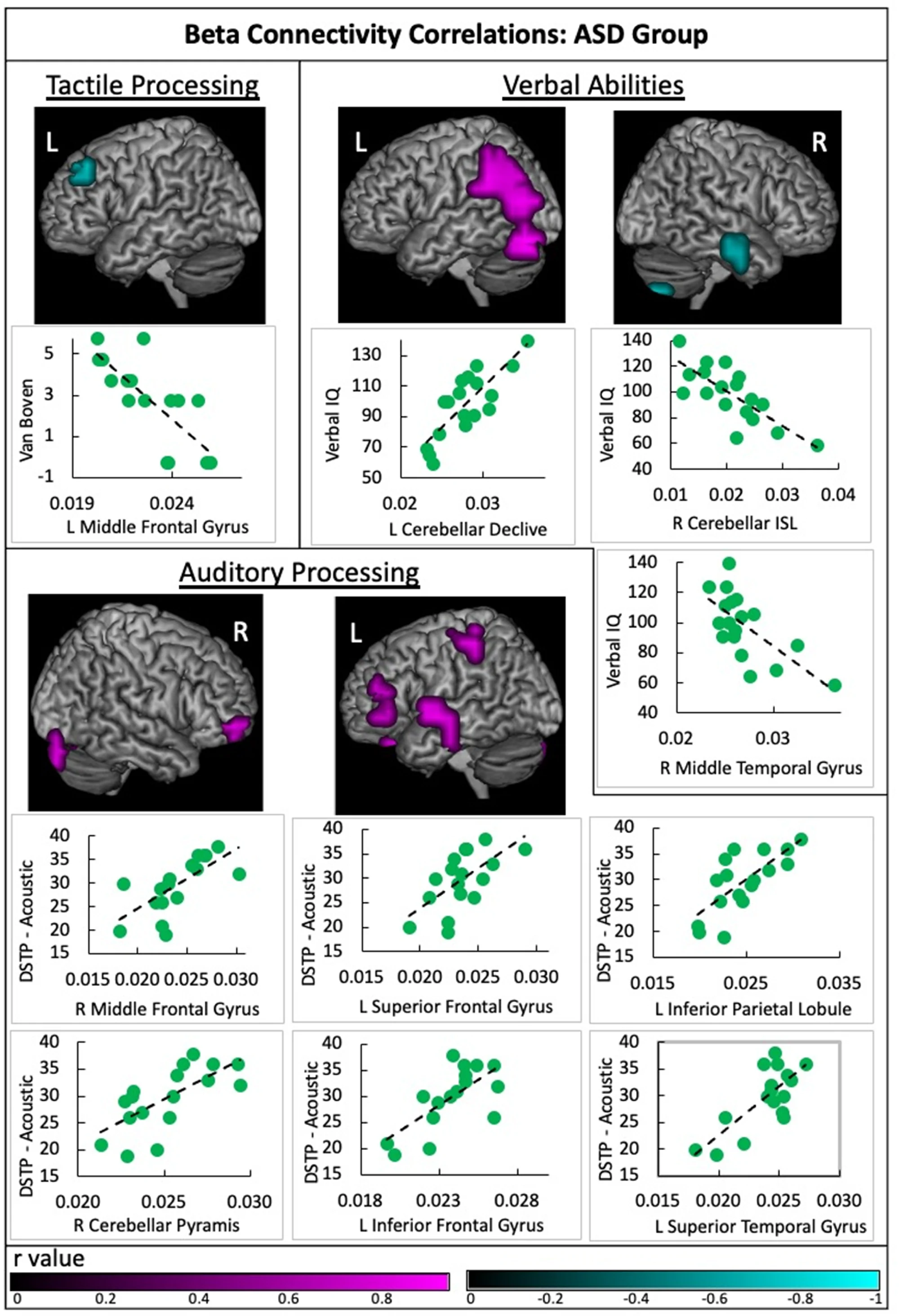
Correlations between clinical measures and beta imaginary coherence in the ASD group. Associations between sensory processing/verbal abilities and imaginary coherence values are identified in clusters (magenta for positive associations and cyan for negative associations). Corresponding scatterplots are presented for the voxel with the maximum correlation value within each cluster. Auditory processing was positively associated beta coherence in a distributed bilateral network, whereas tactile processing was negatively associated with left frontal beta coherence. Verbal abilities showed negative correlations in right temporal and cerebellar regions and positive associations in a large left posterior cluster.

**ASD group beta band correlations.** In the ASD group, tactile processing on the Van Boven Domes task was significantly associated with beta coherence in the left middle frontal gyrus. Auditory processing performance on the DSTP Acoustic Index was significantly associated with beta connectivity in frontal, temporal and parietal brain regions in the left hemisphere and frontal, temporal/occipital and cerebellar structures in the right hemisphere. Both positive and negative associations were identified between beta band imaginary coherence and verbal abilities in the ASD group. Specifically, in the ASD group VIQ was positively associated with beta coherence in a left temporal/parietal/occipital/cerebellar cluster. VIQ was negatively correlated with connectivity in right cerebellar and temporal regions (figure 10).

**Figure 11.**
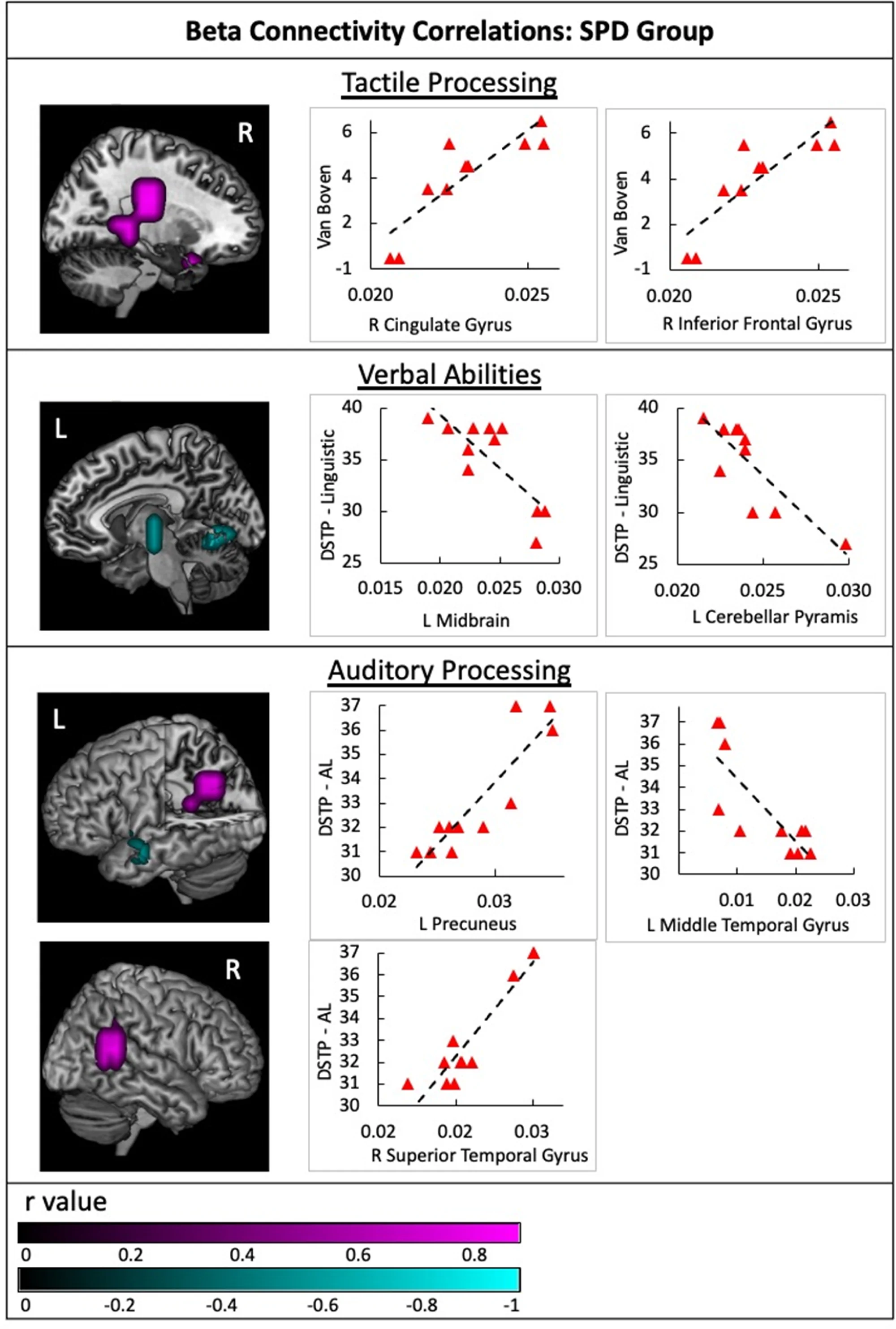
Correlations between clinical measures and beta imaginary coherence in the SPD group. Associations between sensory processing/verbal abilities and imaginary coherence values are identified in clusters (magenta for positive associations and cyan for negative associations). Corresponding scatterplots are presented for the voxel with the greatest correlation value within each cluster. Negative associations were identified between auditory processing and imaginary coherence in left temporal regions and between verbal abilities and mesial subcortical and cerebellar regions. Positive associations were identified between auditory processing and posterior regions and between tactile processing and mesial subcortical regions.

**SPD group beta band correlations.** For the SPD group, tactile processing on the Van Boven Domes task was significantly associated with beta coherence in right medial subcortical structures. Auditory processing on the DSTP Acoustic/Linguistic Index was positively associated with beta coherence in the right parietal/occipital regions and negatively associated with left temporal regions. Performance on the Linguistic Index of the DSTP was negatively associated with left hemisphere beta coherence in the thalamus, midbrain, occipital and cerebellar structures (figure 11).

Relative to TDC, the clinical groups both show increased alpha connectivity in parietal regions, whereas the two clinical groups did not significantly differ from one another. For beta connectivity, however, there was a bilateral difference between clinical groups in IPL and medial SFG connectivity, with ASD > SPD. The SPD group also demonstrated reduced frontal and parietal connectivity relative to the TDC group, suggesting a general pattern of beta underconnectivity in these regions for the SPD participants. In contrast, the ASD group only showed reduced beta connectivity in the left middle and inferior temporal gyri relative to TDC.

Increased alpha cerebellar connectivity was associated with better auditory processing and verbal performance, whereas connectivity in temporal regions showed a differential pattern of reduced alpha connectivity associated with better auditory processing and increased alpha connectivity associated with verbal abilities. With regard to tactile processing, reduced parietal beta connectivity was associated with better tactile performance.

Negative correlations were identified between sensory processing and imaginary coherence in the alpha and beta bands, with associations between auditory processing and left temporal (and occipital for beta) regions and between tactile processing and a more distributed bilateral set of frontal, temporal and parietal regions. In contrast, linguistic performance was positively associated with left frontal alpha coherence.

Positive associations were identified between graphesthesia performance and both right frontal and left temporal and subcortical structures. Auditory processing was positively associated beta coherence in a distributed bilateral network, whereas tactile processing was negatively associated with left frontal beta coherence. Verbal abilities showed negative correlations with beta coherence in right temporal and cerebellar regions and positive associations in a large left posterior cluster.

Negative associations were identified between left temporal alpha and beta coherence and auditory processing and between left cerebellar alpha coherence and Von Boven Domes task performance. Positive associations were identified between Graphesthesia performance and right cerebellar and parietal regions as well as between verbal abilities and a more distributed bilateral network of frontal, temporal, parietal, occipital, and cerebellar regions. Negative associations were identified between verbal abilities and mesial subcortical and cerebellar beta coherence. Positive associations were identified between auditory processing and posterior beta coherence and between tactile processing and mesial subcortical beta coherence.

## 6. Discussion

This study used two methods to investigate associations between direct assessment of auditory and tactile sensory processing and resting state functional connectivity in the brain. First, we examined differences between groups that would allow us to isolate the SPD that presents as part of an ASD from that which manifests in the absence of the other defining features of ASD. Second, we directly examined associations between functional connectivity and auditory and tactile processing and verbal abilities in a combined participant sample including all three groups, allowing us to examine the distribution of these variables across children with a range of sensory functioning.

### 6.1. Group contrasts in imaginary coherence

#### 6.1.1. ASD vs TDC contrasts

Relative to the TDC group, in the alpha band, participants with ASD showed increased imaginary coherence in the right sensorimotor cortex and decreased imaginary coherence in left posterior fusiform, occipital, and cerebellar regions. Notably, increased alpha power (Edgar *et al* 2015b) in a similar region in the right medial sensorimotor cortex, and increased alpha to low-gamma phase amplitude coupling in this central midline region (Port *et al* 2019) has been reported in prior ASD samples. The present results also are in alignment with our previous structural findings in children with ASD, in which we reported decreased structural connectivity in the inferior fronto-occipital fasciculus and the fusiform-hippocampus and fusiform-amygdala tracts (Y.-S. Chang *et al* 2014). Our findings of increased cerebellar connectivity are also consistent with considerable prior research implicating the cerebellum in the presentation of ASD. Specifically, cerebellar anomalies, including abnormal anatomy, neurotransmission, oxidative stress, neuroinflammation, and cerebellar motor and cognitive deficits are among the most replicated findings in individuals with ASD (Fatemi *et al* 2012).

In the beta range, the ASD group demonstrated decreased beta connectivity in left temporal regions relative to TDC participants. Stronger beta connectivity in TDC relative to ASD participants in temporal regions has been demonstrated in prior work (Kitzbichler *et al* 2015). Beta power in the auditory cortex has been hypothesized to be involved in auditory-motor communication (Fujioka *et al* 2009) and recent work has demonstrated increases in sensorimotor low beta power in response to perceived self-produced vocal errors on an altered auditory feedback speech paradigm (Franken *et al* 2018). The decreased beta connectivity in the left auditory cortex demonstrated in the present study is consistent with reduced engagement of auditory cortical networks involved in auditory-motor communication in participants with ASD.

#### 6.1.2. SPD vs TDC contrasts

The SPD group differed from the TDC group via increased alpha connectivity in bilateral frontal and right posterior parietal regions and reduced beta connectivity in left parietal and medial and right frontal regions. These differences in functional connectivity identified in these regions may be associated with the impairments in visuomotor skills and attention previously reported in the SPD population (Brandes-Aitken *et al* 2018). In fact, prior work examining diffusion imaging in children with SPD identified associations between visuomotor and cognitive control abilities and structural connectivity in regions of the superior longitudinal fasciculus that run adjacent to the parietal regions identified in this study (Brandes-Aitken *et al* 2019).

#### 6.1.3. ASD vs SPD alpha and beta contrasts

Notably, the ASD and SPD groups did not show significant differences in alpha connectivity. In fact, it was beta connectivity that distinguished these two groups. Specifically, the SPD group showed a pattern of reduced beta connectivity relative to both the TDC and ASD groups in bilateral and medial frontal and left parietal regions. Taken together, these findings suggest that decreased beta connectivity in medial frontal and parietal regions may represent a neural correlate of the sensory dysfunction observed in children with SPD. Beta activity has been previously reported to be associated with somatosensory gating and attention (van Ede *et al* 2010, Bauer *et al* 2012, Buchholz *et al* 2014). Our previous work has demonstrated common tactile processing deficits in both ASD and SPD groups (Demopoulos *et al* 2015a), although when MEG-acquired somatosensory latencies were compared between these groups, the SPD group demonstrated an intermediate latency and did not significantly differ from TDC or ASD participants (Demopoulos *et al* 2017b). These previous results, in conjunction with the present finding that beta activity distinguished the ASD and SPD groups in the bilateral somatosensory cortex, may suggest that the neurobiology underlying tactile dysfunction in these two groups is divergent.

#### 6.1.4. Combined groups correlation results

When correlation analyses were performed on all participants combined into one group, alpha connectivity was positively associated with auditory and verbal abilities, whereas beta connectivity was negatively associated with tactile processing. Specifically, there was a common area of positive correlation between left cerebellar alpha connectivity and both auditory processing and verbal abilities; however, an additional positive association was identified between left anterior temporal alpha connectivity and verbal abilities. Previous work has identified an association between increased anterior temporal alpha power and autism symptomatology measured via the SRS total score (Cornew *et al* 2012), whereas an additional negative association was identified between posterior temporal alpha connectivity and auditory processing. Taken together, these findings suggest that increased cerebellar alpha connectivity is related to auditory processing and verbal abilities in individuals with ASD (Tecchio *et al* 2003, Gage *et al* 2003a, 2003b, Alcántara *et al* 2004, Oram Cardy *et al* 2005, Oram-Cardy *et al* 2005, Järvinen-Pasley and Heaton 2007, Tomcheck and Dunn 2007, Hitoglou *et al* 2010, DePape *et al* 2012, Edgar *et al* 2013, 2014, 2015a, Abdeltawwab and Baz 2015, Kargas *et al* 2015, Demopoulos *et al* 2015b, 2017b, 2023a, 2023b, Demopoulos and Lewine 2016, Arutiunian *et al* 2023, 2024, 2025, Gallo *et al* 2026). Indeed, prior work has demonstrated links between cortical auditory processing abnormalities and verbal abilities (Oram-Cardy *et al* 2005, Oram Cardy *et al* 2008, Schmidt *et al* 2009, Roberts *et al* 2011, 2012, Edgar *et al* 2013, Berman *et al* 2016, Demopoulos *et al* 2017b). With regard to beta connectivity, increases in the right somatosensory cortex were associated with poorer performance on the graphesthesia task. Examination of the scatterplot distribution suggests that somatosensory processing limitations are consistent with the poorer graphesthesia performance observed in the two clinical groups.

#### 6.1.5. Individual group correlation results

**Tactile Processing.** In the alpha band, associations between functional connectivity and tactile processing in the neurotypical group was bilaterally and broadly represented, reflecting the more diffuse cortical requirement of the graphesthesia task and the administration of the task to the dorsal surface of both hands. Consistent with alpha connectivity correlations, graphesthesia performance in the TDC group also showed diffuse bilateral negative associations, including bilateral frontal regions and a right somatosensory region that was part of a larger cluster identified in the alpha correlations. The consistent negative associations between alpha and beta connectivity and graphesthesia performance suggest that functional connectivity within these distributed networks is related to tactile processing.

In contrast, in our alpha coherence correlational analysis, reduced connectivity in a left temporal and subcortical region including the fusiform gyrus was positively associated with poorer performance on the graphesthesia task in the ASD group. Prior research has demonstrated that the left fusiform gyrus has been associated with identifying letters from lower level shape images (McCandliss *et al* 2003, Dehaene and Cohen 2011). Given the demands of the graphesthesia task involved this type of skill, its positive association with alpha connectivity in the left fusiform gyrus may suggest that pathological under-connectivity of the left fusiform in the alpha band is associated with the difficulty the ASD group demonstrated in perceiving these forms via tactile examination (Demopoulos *et al* 2015a).

**Auditory processing and verbal abilities.** Associations with auditory processing and verbal abilities involved distributed temporal, frontal, and cerebellar regions across both neurodevelopmental groups, consistent with the established involvement of these networks in auditory-language function. Although the specific regional associations differed between groups, these differences should be interpreted cautiously, and the present findings primarily support the broader conclusion that beta-band connectivity within auditory-language networks is related to communication-relevant abilities.

Alpha oscillatory activity has been previously reported to be associated with sensory gating (Buchholz *et al* 2014) and direction of sensory attention in the auditory cortex (Foxe and Snyder 2011). Consistent with these previous findings and classically described linguistic associations, functional connectivity in the alpha oscillatory frequency for our neurotypical pediatric sample was significantly associated with auditory processing in the left temporal lobe (Wernicke’s area) and with linguistic processing in the left inferior frontal lobe (Broca’s area). Also consistent with findings from correlations between alpha connectivity and task data, auditory processing was significantly negatively associated with beta connectivity in Wernicke’s area for the TDC group. These findings extend the alpha-band associations by demonstrating that beta connectivity within left temporal regions was also related to auditory processing.

In the ASD group, increased beta connectivity in left temporal and parietal regions, including auditory and sensorimotor cortex, also were significantly associated with better auditory processing and verbal abilities. Beta power in the auditory cortex has been hypothesized to be involved in auditory-motor communication (Fujioka *et al* 2009) and recent work has demonstrated increases in sensorimotor low beta power in response to perceived self-produced vocal errors on an altered auditory feedback speech paradigm (Franken *et al* 2018). The positive relationships between left temporal and parietal beta connectivity and auditory and verbal abilities demonstrated in the present study are consistent with a role for beta connectivity in auditory-motor communication.

#### 6.1.6. Limitations and future directions

Several limitations of the present study must be acknowledged. First, the participant sample was restricted to males between the ages of 8–12 years. Prior studies examining resting state neural oscillatory behavior have also restricted analyses to males given the higher prevalence of ASD in males (Zeidan *et al* 2022) and sex differences in peak alpha frequency (Edgar *et al* 2019, Green *et al* 2022, Manyukhina *et al* 2022). While these restrictions result in more homogenous groups and minimize confounds of sex and age differences in neurobiology, they also create limitations for the generalizability of these results to females and children and adolescents outside the age range studied. Future research with larger samples including males and females can study the effects of sex differences on these relationships directly. This study also included only children with a nonverbal IQ > 70 in order to isolate the neurobiological differences associated with autism from those associated with autism with co-occurring intellectual disability, which limits the generalizability of these results to individuals with intellectual disability. Future research is necessary to understand the applicability of these findings across ages, sexes and cognitive abilities.

Second, left-handed and ambidextrous participants were included, and these participants may have been more likely to have differences in functional lateralization, which we were unable to assess and control for in the present study. Future research should include assessment of functional lateralization so that it can be included in the study design to measure the effects of differences in lateralization on neural behavior and sensory dysfunction. Further, this study focused on only two sensory domains, auditory and tactile processing. While prior research suggests that these domains may be the most severely impacted in individuals with ASD (Fernandez-Andres *et al* 2015), sensory dysfunction is heterogeneous in its presentation among individuals with and without ASD, and understanding neurobiological factors associated with dysfunction in other sensory domains also will be important to inform treatment development.

Diagnoses were also made based on DSM-IV-TR criteria. As such, results may not generalize to groups assessed according to DSM-5 or future evolving diagnostic criteria. Future research should also examine the presentation of sensory dysfunction and its neural correlates in other groups for which sensory processing may be impacted, such as individuals with attention-deficit/hyperactivity disorder (ADHD). Studies that cross diagnostic boundaries of these behaviorally-defined disorders will allow us to better understand the similarities and differences in the neurobiology contributing to sensory dysfunction as well as the impact of sensory dysfunction on other domains of functioning, such as attention.

Additionally, this study focused on specific aspects of sensory processing (e.g. discrimination, temporal processing, etc), but did not incorporate measures of sensory responsivity or sensory seeking behavior. Further, this work only focused on two frequency bands, alpha and beta. Future studies could expand upon this work to examine relations between SPD and functional connectivity in other frequency bands, as gamma oscillatory behavior has been associated with multisensory communication (Misselhorn *et al* 2019) and sensory sensitivity (Manyukhina *et al* 2021). Future studies are needed to characterize differences in functional connectivity that may account for these heterogeneous sensory responses or behaviors in children with ASD and SPD.

Participants in this study sample also were taking multiple different medications. Our post hoc analysis controlling for medication usage indicated that, while most findings remained statistically significant after controlling for class of drugs use, one contrast was no longer statistically significant after controlling for medication usage. This highlights the importance of understanding the influence of different medications on brain activity and connectivity. Future research is needed that includes detailed assessment of medication usage, including, type, dosage, and duration of use, in order to understand the impact of medications on brain imaging results.

Finally, the SPD group was underrepresented in the correlation analyses between functional connectivity and sensory behavioral tasks. As such, these results only provide preliminary evidence of differences in neurobiology of individuals without ASD who experience sensory dysfunction. Future large scale studies are needed to adequately control for all of these factors in order to better understand the neurobiology of sensory dysfunction and to determine in results presented in the extant literature are replicable.

## 7. Conclusions

This study was the first to use MEG to examine participants with ASD and SPD in relation to neurotypical children to identify relevant differences in resting state whole brain functional connectivity that may be associated with sensory dysfunction. This study design allowed us to identify both shared and distinct patterns of neural activity in two groups affected by sensory dysfunction. Specifically, both clinical groups were distinguished from the TDC group by patterns of functional connectivity differences in the alpha and beta bands, whereas the clinical groups were only distinguished from each other on measures of beta connectivity. Associations between functional connectivity and behavior varied across groups, suggesting differences in neural processes underlying sensory processing and verbal abilities in children with ASD and SPD relative to TDC youth. Thus, these results suggest that resting state differences in oscillatory brain activity in the alpha and beta frequencies may be associated with the sensory dysfunction that characterizes ASD and SPD.

## Data Availability

The data cannot be made publicly available upon publication because they are not available in a format that is sufficiently accessible or reusable by other researchers. The data that support the findings of this study are available upon reasonable request from the authors.

## Data access statement

Data will be made available upon reasonable request to EJM.

## References

[jneae93fbbib1] Abdeltawwab M M, Baz H (2015). Automatic pre-attentive auditory responses: MMN to tone burst frequency changes in autistic school-age children. J. Int. Adv. Otol..

[jneae93fbbib2] Adamson A, Hare A O, Graham C (2006). Impairments in sensory modulation in children with autistic spectrum disorder. Br. J. Occup. Ther..

[jneae93fbbib3] Adolph D, Margraf J (2017). The differential relationship between trait anxiety, depression, and resting frontal α-asymmetry. J. Neural Transm..

[jneae93fbbib4] Ahn R R, Miller L J, Milberger S, McIntosh D N (2004). Prevalence of parents’ perceptions of sensory processing disorders among kindergarten children. Am. J. Occup. Ther..

[jneae93fbbib5] Alcántara J I, Weisblatt E J L, Moore B C J, Bolton P F (2004). Speech-in-noise perception in high-functioning individuals with autism or Asperger’s syndrome. J. Child Psychol. Psychiatry Allied Discip..

[jneae93fbbib6] Al-Heizan M O, AlAbdulwahab S S, Kachanathu S J, Natho M (2015). Sensory processing dysfunction among Saudi children with and without autism. J. Phys. Ther. Sci..

[jneae93fbbib7] American Psychiatric Association (2013). Diagnostic and Statistical Manual of Mental Disorders.

[jneae93fbbib8] Arutiunian V, Arcara G, Buyanova I, Davydova E, Pereverzeva D, Sorokin A, Tyushkevich S, Mamokhina U, Danilina K, Dragoy O (2023). Neuromagnetic 40 Hz auditory steady-state response in the left auditory cortex is related to language comprehension in children with autism spectrum disorder. Prog. Neuropsychopharmacol. Biol. Psychiatry.

[jneae93fbbib9] Arutiunian V (2024). Abnormalities in both stimulus-induced and baseline MEG alpha oscillations in the auditory cortex of children with Autism Spectrum Disorder. Brain Struct. Funct..

[jneae93fbbib10] Arutiunian V, Buyanova I, Minnigulova A, Davydova E, Pereverzeva D, Sorokin A, Tyushkevich S, Mamokhina U, Danilina K, Dragoy O (2025). Left-hemispheric atypicalities in the primary auditory cortex are associated with language comprehension and social skills in children with Autism Spectrum Disorder. Cereb. Cortex.

[jneae93fbbib11] Ayres J (1989). Sensory Integration and Praxis Tests (SIPT).

[jneae93fbbib12] Bauer M, Kennett S, Driver J (2012). Attentional selection of location and modality in vision and touch modulates low-frequency activity in associated sensory cortices. J. Neurophysiol..

[jneae93fbbib13] Benjamini Y, Hochberg Y (1995). Controlling the false discovery rate: a practical and powerful approach to multiple testing. J. R. Stat. Soc..

[jneae93fbbib14] Berman J, Liu S, Bloy L, Blaskey L, Roberts T, Edgar J (2015). Alpha-to-gamma phase-amplitude coupling methods and application to autism spectrum disorder. Brain Connect..

[jneae93fbbib15] Berman J I, Edgar J C, Blaskey L, Kuschner E S, Levy S E, Ku M, Dell J, Roberts T P L (2016). Multimodal diffusion-MRI and MEG assessment of auditory and language system development in autism spectrum disorder. Front. Neuroanat..

[jneae93fbbib16] Boutcher S H, Landers D M (1998). The effects of vigorous exercise on anxiety, heart rate, and alpha activity of runners and nonrunners. Psychophysiology.

[jneae93fbbib17] Brandes-Aitken A, Anguera J A, Chang Y-S, Demopoulos C, Owen J P, Gazzaley A, Mukherjee P, Marco E J (2019). White matter microstructure associations of cognitive and visuomotor control in children: a sensory processing perspective. Front. Integr. Neurosci..

[jneae93fbbib18] Brandes-Aitken A, Anguera J A, Rolle C E, Desai S S, Demopoulos C, Skinner S N, Gazzaley A, Marco E J (2018). Characterizing cognitive and visuomotor control in children with sensory processing dysfunction and autism spectrum disorders. Neuropsychology.

[jneae93fbbib19] Brodski-Guerniero A (2018). Predictable information in neural signals during resting state is reduced in autism spectrum disorder. Hum. Brain Mapp..

[jneae93fbbib20] Buchholz V N, Jensen O, Medendorp W P (2014). Different roles of alpha and beta band oscillations in anticipatory sensorimotor gating. Front. Hum. Neurosci..

[jneae93fbbib21] Chang Y S, Gratiot M, Owen J P, Brandes-Aitken A, Desai S S, Hill S S, Arnett A B, Harris J, Marco E J, Mukherjee P (2016). White matter microstructure is associated with auditory and tactile processing in children with and without sensory processing disorder. Front. Neuroanat..

[jneae93fbbib22] Chang Y-S, Owen J P, Desai S S, Hill S S, Arnett A B, Harris J, Marco E J, Mukherjee P (2014). Autism and sensory processing disorders: shared white matter disruption in sensory pathways but divergent connectivity in social-emotional pathways. PLoS One.

[jneae93fbbib23] Cornew L, Roberts T P L, Blaskey L, Edgar J C (2012). Resting-state oscillatory activity in autism spectrum disorders. J. Autism Dev. Disord..

[jneae93fbbib24] Dalal S S, Zumer J M, Guggisberg A G, Trumpis M, Wong D D E, Sekihara K, Nagarajan S S (2011). MEG/EEG source reconstruction, statistical evaluation, and visualization with NUTMEG. Comput. Intell. Neurosci..

[jneae93fbbib25] Dehaene S, Cohen L (2011). The unique role of the visual word form area in reading. Trends Cogn. Sci..

[jneae93fbbib26] Demerdzieva A, Pop-jordanova N (2015). Relation between frontal alpha asymmetry and anxiety in young patients with generalized anxiety disorder. Prilozi.

[jneae93fbbib27] Demopoulos C (2017a). Magnetoencephalographic imaging of auditory and somatosensory cortical responses in children with autism and sensory processing dysfunction. Front. Hum. Neurosci..

[jneae93fbbib28] Demopoulos C, Brandes-Aitken A N, Desai S S, Hill S S, Antovich A D, Harris J, Marco E J (2015a). Shared and divergent auditory and tactile processing in children with autism and children with sensory processing dysfunction relative to typically developing peers. J. Int. Neuropsychol. Soc..

[jneae93fbbib29] Demopoulos C (2020). Global resting-state functional connectivity of neural oscillations in tinnitus with and without hearing loss. Hum. Brain Mapp..

[jneae93fbbib30] Demopoulos C, Hopkins J, Kopald B E B E, Paulson K, Doyle L, Andrews W E W E, Lewine J D J D (2015b). Deficits in auditory processing contribute to impairments in vocal affect recognition in autism spectrum disorders: a MEG study. Neuropsychology.

[jneae93fbbib31] Demopoulos C, Kopald B E, Bangera N, Paulson K, Lewine J D (2023a). Rapid auditory processing of puretones is associated with basic components of language in individuals with autism spectrum disorders. Brain Lang..

[jneae93fbbib32] Demopoulos C, Lewine J D J D (2016). Audiometric profiles in autism spectrum disorders: does subclinical hearing loss impact communication?. Autism Res..

[jneae93fbbib33] Demopoulos C, Skiba S A, Kopald B E, Bangera N, Paulson K, Lewine J D (2023b). Associations between rapid auditory processing of speech sounds and specific verbal communication skills in autism. Front. Psychol..

[jneae93fbbib34] Demopoulos C (2017b). Magnetoencephalographic imaging of auditory and somatosensory cortical responses in children with autism and sensory processing dysfunction. Front. Hum. Neurosci..

[jneae93fbbib35] DePape A-M R, Hall G B C, Tillmann B, Trainor L J (2012). Auditory processing in high-functioning adolescents with autism spectrum disorder. PLoS One.

[jneae93fbbib36] Dunn W (1999). Sensory Profile User’s Manual.

[jneae93fbbib37] Edgar J C (2019). Abnormal maturation of the resting-state peak alpha frequency in children with autism spectrum disorder. Hum. Brain Mapp..

[jneae93fbbib38] Edgar J C, Fisk IV C L, Berman J I, Chudnovskaya D, Liu S, Pandey J, Herrington J D, Port R G, Schultz R T, Roberts T P L (2015a). Auditory encoding abnormalities in children with autism spectrum disorder suggest delayed development of auditory cortex. Mol. Autism.

[jneae93fbbib39] Edgar J C (2015b). Resting-state alpha in autism spectrum disorder and alpha associations with thalamic volume. J. Autism Dev. Disord..

[jneae93fbbib40] Edgar J C (2013). Neuromagnetic oscillations predict evoked-response latency delays and core language deficits in autism spectrum disorders. J. Autism Dev. Disord..

[jneae93fbbib41] Edgar J C (2014). Missing and delayed auditory responses in young and older children with autism spectrum disorders. Front. Hum. Neurosci..

[jneae93fbbib42] Eigsti I M, Schuh J M (2008). Neurobiological underpinnings of language in autism spectrum disorders. Annu. Rev. Appl. Linguist..

[jneae93fbbib43] Engel A K, Gerloff C, Hilgetag C C, Nolte G (2013). Intrinsic coupling modes: multiscale interactions in ongoing brain activity. Neuron.

[jneae93fbbib44] Fatemi S H (2012). Consensus paper: pathological role of the cerebellum in Autism. Cerebellum.

[jneae93fbbib45] Fernandez-Andres M I, Pastor-Cerezuela G, Sanz-Cervera P, Tarraga-Mingues R (2015). A comparative study of sensory processing in children with and without autism spectrum disorder in the home and classroom environments. Res. Dev. Disabil..

[jneae93fbbib46] Fingelkurts A A, Fingelkurts A A, Rytsälä H, Suominen K, Isometsä E, Kähkönen S (2007). Impaired functional connectivity at EEG alpha and theta frequency bands in major depression. Hum. Brain Mapp..

[jneae93fbbib47] Foxe J J, Snyder A C (2011). The role of alpha-band brain oscillations as a sensory suppression mechanism during selective attention. Front. Psychol..

[jneae93fbbib48] Franken M K, Eisner F, Acheson D J, McQueen J M, Hagoort P, Schoffelen J M (2018). Self-monitoring in the cerebral cortex: neural responses to small pitch shifts in auditory feedback during speech production. NeuroImage.

[jneae93fbbib49] Fujioka T, Trainor L J, Large E W, Ross B (2009). Beta and gamma rhythms in human auditory cortex during musical beat processing. Ann. New York Acad. Sci..

[jneae93fbbib50] Gage N M, Siegel B, Callen M, Roberts T (2003a). Cortical sound processing in children with autism disorder : an MEG investigation. Neuroreport.

[jneae93fbbib51] Gage N M, Siegel B, Roberts T P L (2003b). Cortical auditory system maturational abnormalities in children with autism disorder: an MEG investigation. Dev. Brain Res..

[jneae93fbbib52] Gallo A, Sabes J H, Demopoulos C (2026). Auditory processing and communication in autism: exploring verbal abilities and vocal affective cues. Front. Psychiatry.

[jneae93fbbib53] Green H L (2022). Peak alpha frequency and thalamic structure in children with typical development and autism spectrum disorder. J. Autism Dev. Disord..

[jneae93fbbib54] Green H L, Edgar J C, Matsuzaki J, Roberts T P L (2020). Magnetoencephalography research in pediatric autism spectrum disorder. Neuroimaging Clin. North Am..

[jneae93fbbib55] Greenspan S I, Wieder S (1997). Developmental patterns and outcomes in infants and children with disorders in relating and communicating: a chart review of 200 cases of children with autistic spectrum diagnoses. J. Dev. Learn. Disord..

[jneae93fbbib56] Guggisberg A G, Honma S M, Findlay A M, Dalal S S, Kirsch H E, Berger M S, Nagarajan S S (2008). Mapping functional connectivity in patients with brain lesions. Ann. Neurol..

[jneae93fbbib57] Hinkley L (2010). Cognitive impairments in schizophrenia as assessed through activation and connectivity measures of magnetoencephalography (MEG) data. Front. Hum. Neurosci..

[jneae93fbbib58] Hinkley L, Vinogradov S, Guggisberg A G, Fisher M, Findlay A M, Nagarajan S S (2011). Clinical symptoms and alpha band resting-state functional connectivity imaging in patients with schizophrenia: implications for novel approaches to treatment. Biol. Psychiatry.

[jneae93fbbib59] Hitoglou M, Ververi A, Antoniadis A, Zafeiriou D I (2010). Childhood autism and auditory system abnormalities. Pediatr. Neurol..

[jneae93fbbib60] Järvinen-Pasley A, Heaton P (2007). Evidence for reduced domain-specificity in auditory processing in autism. Dev. Sci..

[jneae93fbbib61] Kargas N, Lopez B, Reddy V, Morris P, López B, Reddy V, Morris P (2015). The relationship between auditory processing and restricted, repetitive behaviors in adults with autism spectrum disorders. J. Autism Dev. Disord..

[jneae93fbbib62] Kitzbichler M G, Khan S, Ganesan S, Vangel M G, Herbert M R, Hämäläinen M S, Kenet T (2015). Altered development and multifaceted band-specific abnormalities of resting state networks in autism. Biol. Psychiatry.

[jneae93fbbib63] Knyazev G G, Savostyanov A N, Levin E A (2006). Alpha synchronization and anxiety: implications for inhibition vs. alertness hypotheses. Int. J. Psychophysiol..

[jneae93fbbib64] Lerner M D, McPartland J C, Morris J P (2013). Multimodal emotion processing in autism spectrum disorders: an event-related potential study. Dev. Cogn. Neurosci..

[jneae93fbbib65] Li J, Sujawal M, Bernotaite Z, Cunnings I, Liu F (2025). Auditory and Semantic Processing of Speech-in-Noise in Autism: a Behavioral and EEG Study. Autism Res..

[jneae93fbbib66] Lord C, Rutter M, Goode S, Heemsbergen J, Jordan H, Mawhood L, Schopler E (1989). Autism diagnostic observation schedule: a standardized observation of communicative and social behavior. J. Autism Dev. Disord..

[jneae93fbbib67] Lord C, Rutter M, Le Couteur A (1994). Autism Diagnostic Interview-Revised: a revised version of a diagnostic interview for caregivers of individuals with possible pervasive developmental disorders. J. Autism Dev. Disord..

[jneae93fbbib68] Manyukhina V O, Prokofyev A O, Galuta I A, Goiaeva D E, Obukhova T S, Schneiderman J F, Altukhov D I, Stroganova T A, Orekhova E V (2022). Globally elevated excitation–inhibition ratio in children with autism spectrum disorder and below-average intelligence. Mol. Autism.

[jneae93fbbib69] Manyukhina V O, Rostovtseva E N, Prokofyev A O, Obukhova T S, Schneiderman J F, Stroganova T A, Orekhova E V (2021). Visual gamma oscillations predict sensory sensitivity in females as they do in males. Sci. Rep..

[jneae93fbbib70] Martino J, Honma S M, Findlay A M, Guggisberg A G, Owen J P, Kirsch H E, Berger M S, Nagarajan S S (2011). Resting functional connectivity in patients with brain tumors in eloquent areas. Ann. Neurol..

[jneae93fbbib71] Mayes S D, Calhoun S L (1999). Symptoms of autism in young children and correspondence with the DSM. Infants Young Child..

[jneae93fbbib72] McCandliss B D, Cohen L, Dehaene S (2003). The visual word form area: expertise for reading in the fusiform gyrus. Trends Cogn. Sci..

[jneae93fbbib73] Mennella R, Patron E, Palomba D (2017). Frontal alpha asymmetry neurofeedback for the reduction of negative affect and anxiety. Behav. Res. Ther..

[jneae93fbbib74] Misselhorn J, Schwab B C, Schneider T R, Engel A K (2019). Synchronization of sensory gamma oscillations promotes multisensory communication. ENeuro.

[jneae93fbbib75] Nolte G, Bai O, Wheaton L, Mari Z, Vorbach S, Hallett M (2004). Identifying true brain interaction from EEG data using the imaginary part of coherency. Clin. Neurophysiol..

[jneae93fbbib76] Oram Cardy J E, Flagg E J, Roberts W, Roberts T P L (2005). Delayed mismatch field for speech and non-speech sounds in children with autism. Neuroreport.

[jneae93fbbib77] Oram Cardy J E, Flagg E J, Roberts W, Roberts T P L (2008). Auditory evoked fields predict language ability and impairment in children. Int. J. Psychophysiol..

[jneae93fbbib78] Oram-Cardy J E, Flagg C A E J, Roberts W, Brian J, Roberts T P L (2005). Magnetoencephalography identifies rapid temporal processing deficit in autism and language impairment. Neuroreport.

[jneae93fbbib79] Owen J P, Marco E J, Desai S, Fourie E, Harris J, Hill S S, Arnett A B, Mukherjee P (2013). Abnormal white matter microstructure in children with sensory processing disorders. Neuroimage Clin..

[jneae93fbbib80] Papanicolaou A C (2009). Clinical Magnetoencephalography and Magnetic Source Imaging.

[jneae93fbbib81] Port R G, Dipiero M A, Ku M, Liu S, Blaskey L, Kuschner E S, Edgar J C, Roberts T P L, Berman J I (2019). Children with autism spectrum disorder demonstrate regionally specific altered resting-state phase-amplitude coupling. Brain Connect..

[jneae93fbbib82] Ranasinghe K G (2017). Distinct spatiotemporal patterns of neuronal functional connectivity in primary progressive aphasia variants. Brain.

[jneae93fbbib83] Richard G J, Ferre J M (2006). Differential screening test for processing.

[jneae93fbbib84] Roberts T P L, Cannon K M, Tavabi K, Blaskey L, Khan S Y, Monroe J F, Qasmieh S, Levy S E, Edgar J C (2011). Auditory magnetic mismatch field latency: a biomarker for language impairment in autism. Biol. Psychiatry.

[jneae93fbbib85] Roberts T P L, Heiken K, Kahn S Y, Qasmieh S, Blaskey L, Solot C, Parker W A, Verma R, Edgar J C (2012). Delayed magnetic mismatch negativity field, but not auditory M100 response, in specific language impairment. Neuroreport.

[jneae93fbbib86] Roberts T P L (2010). MEG detection of delayed auditory evoked responses in autism spectrum disorders: towards an imaging biomarker for autism. Autism Res..

[jneae93fbbib87] Roberts T P L, Matsuzaki J, Blaskey L, Bloy L, Edgar J C, Kim M, Ku M, Kuschner E S, Embick D (2019). Delayed M50/M100 evoked response component latency in minimally verbal/nonverbal children who have autism spectrum disorder. Mol. Autism.

[jneae93fbbib88] Roberts T P L, Schmidt G L, Egeth M, Blaskey L, Rey M M, Edgar J C, Levy S E (2008). Electrophysiological signatures: magnetoencephalographic studies of the neural correlates of language impairment in autism spectrum disorders. Int. J. Psychophysiol..

[jneae93fbbib89] Russo N, Zecker S, Trommer B, Chen J, Kraus N (2009). Effects of background noise on cortical encoding of speech in autism spectrum disorders. J. Autism Dev. Disord..

[jneae93fbbib90] Rutter M, Bailey A, Lord C (2003). SCQ: Social Communication Questionnaire.

[jneae93fbbib91] Schmidt G L, Rey M M, Oram Cardy J E, Roberts T P L (2009). Absence of M100 source asymmetry in autism associated with language functioning. Neuroreport.

[jneae93fbbib92] Schwartz S, Shinn-Cunningham B, Tager-Flusberg H (2018). Meta-analysis and systematic review of the literature characterizing auditory mismatch negativity in individuals with autism. Neurosci. Biobehav. Rev..

[jneae93fbbib93] Schwartz S, Wang L, Uribe S, Shinn-Cunningham B G, Tager-Flusberg H (2023). Auditory evoked potentials in adolescents with autism: an investigation of brain development, intellectual impairment, and neural encoding. Autism Res..

[jneae93fbbib94] Shibasaki H, Miyazaki M (1992). Event-related potential studies in infants and children. J. Clin. Neurophysiol..

[jneae93fbbib95] Smith E E, Zambrano-Vazquez L, Allen J J B (2016). Patterns of alpha asymmetry in those with elevated worry, trait anxiety, and obsessive-compulsive symptoms: a test of the worry and avoidance models of alpha asymmetry. Neuropsychologia.

[jneae93fbbib96] Tecchio F, Benassi F, Zappasodi F, Gialloreti L E, Palermo M, Seri S, Rossini P M (2003). Auditory sensory processing in autism: a magnetoencephalographic study. Biol. Psychiatry.

[jneae93fbbib97] Tomcheck S, Dunn W (2007). Sensory processing in children with and without autism: a comparative study using the short sensory profile. Am. J. Occup. Ther..

[jneae93fbbib98] *Van Boven Domes* (n.d.).

[jneae93fbbib99] Van Boven R W, Johnson K O (1994). The limit of tactile spatial resolution in humans: grating orientation discrimination at the lip, tongue, and finger. Neurology.

[jneae93fbbib100] van Ede F, Jensen O, Maris E (2010). Tactile expectation modulates pre-stimulus Α-band oscillations in human sensorimotor cortex. NeuroImage.

[jneae93fbbib101] Wechsler D (2003). Wechsler Intelligence Scale for Children-Fourth Edition (WISC-IV).

[jneae93fbbib102] Zeidan J, Fombonne E, Scorah J, Ibrahim A, Durkin M S, Saxena S, Yusuf A, Shih A, Elsabbagh M (2022). Global prevalence of autism: a systematic review update. Autism Res..

